# Time-varying trends from Arctic ozonesonde time series in the years 1994–2022

**DOI:** 10.1038/s41598-024-75364-7

**Published:** 2024-11-12

**Authors:** K. Nilsen, R. Kivi, M. Laine, D. Poyraz, R. Van Malderen, P. von der Gathen, D. W. Tarasick, L. Thölix, N. Jepsen

**Affiliations:** 1 Sodankylä Geophysical Observatory, University of Oulu, 99600 Sodankylä, Finland; 2 Space and Earth Observation Centre, Finnish Meteorological Institute, 99600 Sodankylä, Finland; 3 Meteorological and Marine Research Programme, Finnish Meteorological Institute, 00560 Helsinki, Finland; 4 Scientific Service Observations, Royal Meteorological Institute and Solar-Terrestrial Centre of Excellence, 1180 Uccle, Belgium; 5 Alfred Wegener Institute, Helmholtz Centre for Polar and Marine Research, 14473 Potsdam, Germany; 6 Air Quality Research Division, Environment and Climate Change Canada, Ontario, ON M3H 5T4 Canada; 7 Climate Research Programme, Finnish Meteorological Institute, 00560 Helsinki, Finland; 8 Research and Development, Danish Meteorological Institute, 2100 Copenhagen, Denmark

**Keywords:** Long-term trends in Arctic ozone, Troposphere, Lower stratosphere, Environmental sciences, Physics

## Abstract

Although evidence of recovery in Antarctic stratospheric ozone has been found, evidence of recovery in Arctic ozone is still elusive, even though 25 years have passed since the peak in ozone depleting substances. Here we have used a Dynamic Linear Model to derive time-varying trends over 20-year periods in the Arctic ozone time series, measured in-situ by ozonesondes from 6 stations, from 1994 to 2022. The model accounts for seasonality, external forcing and 1st-order correlation in the residuals. As proxies for the external forcing, we have used tropopause pressure (replaced with Arctic Oscillation in the troposphere), eddy heat flux, the volume of polar stratospheric clouds multiplied by effective equivalent stratospheric chlorine, and solar radio flux at 10.7 cm for the 11-year solar cycle. Our results indicate that the ozone recovery in the lower Arctic stratosphere is not detectable. Though significant positive trends have been detected prior to 2017 at some stations, there are no statistically significant positive trends after 2017. Moreover, at a number of stations the trends after 2019 are rather negative and significant, varying between − 0.30 ± 0.25 and − 1.00 ± 0.85% per decade. Furthermore, the Arctic troposphere exhibited only statistically significant negative trends over 20-year periods ending in 2017 or later, varying between − 0.31 ± 0.27 and − 1.76 ± 0.41% per decade. These results highlight the importance of continued monitoring of the Arctic ozone.

## Introduction

Despite being a minor constituent in the Earth’s atmosphere, ozone effectively absorbs most of the harmful UV radiation from the Sun, thus protecting the life on Earth^1^. Atmospheric ozone studies became a major concern when Farman et al.^2^ discovered the stratospheric ozone hole above Antarctica during the spring in the year 1985. Anthropogenic Ozone Depleting Substances (ODS), such as chlorofluorocarbons (CFCs), are dissociated by UV radiation above the tropical ozone layer^3^. In this process the chlorine is released, with the potential to destroy ozone, but it is rapidly captured in the reservoir gases $\textrm{HCl}$ and $\mathrm {ClONO_2}$. These species are then transported to the polar region over time. During polar winter night conditions, a vortex is formed in the stratosphere. The isolated air mass in the polar vortex cools down to temperatures that allow the formation of Polar Stratospheric Clouds (PSC). The $\textrm{HCl}$ and $\mathrm {ClONO_2}$ species react heterogeneously on the surface of the PSC. This liberates chlorine, which destroys ozone in catalytic cycles once sunlight returns in spring. In 1987, the Montreal Protocol was initiated and began a process toward banning ODS to prevent further harm to the ozone layer. Since then, a substantial scientific effort has been made to study long-term trends in ozone to find evidence of recovery following the decline of anthropogenic ODS.

Polar stratospheric levels of ODS peaked around the year 2000, and have since then declined by about 25%^4^. Robust evidence of recovery in Antarctic stratospheric ozone has been found^4,5^, although the ozone hole above the Antarctic is still a recurring phenomenon, and large, long-lived ozone holes have occurred recently, likely as a result of dynamical changes^6^. In the Arctic, higher dynamical variability^7^ causes the Arctic vortex to be warmer and much less stable than the Antarctic vortex, resulting in weaker depletion and stronger inter-annual variations of Arctic ozone. This makes it more challenging to detect long-term trends, compared to the Antarctic^4^.

As stratospheric levels of the ODS have been declining since the year 2000, a recovery in Arctic ozone is expected. However, Santer et al.^8^ provide experimental confirmation of ongoing climate change due to increased anthropogenic Green House Gases (GHG), which causes cooling of the stratosphere over time. For the Arctic stratosphere, studies^9–15^ have shown that an increase in anthropogenic GHG emissions and the subsequent cooling of the stratosphere lead to conditions that favor increased formation of PSCs, which is correlated with strong chemical loss of ozone. For example, in the years 2011^16^ and 2020^17^, low dynamical activity in the Arctic provided the conditions to maintain prolonged ODS activation. This caused chemical depletion in Arctic stratospheric ozone with a magnitude similar to the ozone hole seen above the Antarctic^16,17^, despite the already declining levels of ODS. Furthermore, certain scenarios of model-based projections by von der Gathen et al.^15^ suggest that the large Arctic ozone losses could persist or even worsen toward the end of this century if anthropogenic GHG continue to increase steeply over time. As a consequence, despite the decline in stratospheric levels of ODS over time, a recovery in Arctic ozone may not be expected in the near future.

The global warming of the troposphere is caused by anthropogenic emissions of carbon dioxide and other trace gases such as ozone^18–21^. However, for the Arctic, the warming is more rapid compared to the rest of the globe^20,21^. Additionally, unprecedented ozone losses in the northern extra-tropical troposphere have occurred as well. During the spring and summer of 2020, ozonesondes recorded an unusually low ozone content in the northern extra-tropical troposphere, which in most part was due to COVID-19-related reductions in anthropogenic emission of ozone precursor species, but also in minor part because of the 2020 springtime ozone depletion of the Arctic stratosphere^22^.

Tarasick et al.^23^ used a simple linear regression model to derive a trend in Arctic ozone above Resolute in the years 1966–2013 and found a 5% decline in the lower stratosphere. Following the decline of the ODS, a recovery in Arctic ozone is expected at a rate of roughly 3–4 times smaller in magnitude compared to the preceding decline^4,24^. Recent studies on long-term trends from the total column of Arctic ozone have found potential sign of recovery. For example, Bernet et al.^25^ analyzed monthly trends in the period 2000–2020 and reported a positive but not significant March trend, which they consider as a possible indication of springtime recovery in Arctic ozone. Another study by Pazmino et al.^26^ analyzed long-term trends in three different metrics for the period 2000–2021. In one metric, which is based on ozone loss anomaly with respect to sunlit Volume of the Polar Stratospheric Clouds (VPSC) within the polar vortex, they found a negative trend that indicates a reduction in ozone loss, thus an indication of recovery in Arctic total ozone column.

In this paper, we report time-varying trends over 20-year periods in ozonesonde time series from 6 Arctic stations, i.e. Alert, Eureka, Ittoqqortoormiit, Ny-Ålesund, Resolute, and Sodankylä (see Table 1 or Fig. S1 for geographic locations), in the years 1994–2022. The data from the ozonesondes are prepared into monthly and height-averaged times series in which the altitude layers are $\mathrm {L_1}$ (surface–tropopause), $\mathrm {L_2}$ (tropopause—150 hPa), $\mathrm {L_3}$ (150–40 hPa) and $\mathrm {L_4}$ (40–15 hPa). The trends are derived from the Dynamic Linear Model (DLM, see Methods). This model is based on a state space framework and hierarchical Bayesian approach, which allows it to capture trends that can change over time (i.e., non-linear), without the need to assume an inflection point in time as required for piece-wise linear regression model^27–29^. This is advantageous for detecting trend in time series of atmospheric ozone, as it can be expected to be non-linear^27,28,30^. Additionally, this model accounts for seasonality, the effect of external forcers and removes $\mathrm {1^{st}}$ order correlation by adding noise. For the external forcings, we have used five known process-oriented proxies for the Arctic ozone (see Methods). The proxies are: Volume of Polar Stratospheric Clouds multiplied with Effective Equivalent Stratospheric Chlorine (VPSC*EESC), Eddy Heat Flux (EHF), Solar radio Flux 10.7cm (SF) and Tropopause Pressure (TP), which is replaced by Arctic Oscillation (AO) in the troposphere ($\mathrm {L_1}$). The EESC is a convenient measure to estimate the impact of ozone depleting stratospheric chlorine and bromine levels in the Arctic stratosphere^31^.

## Results

**Figure 1:**
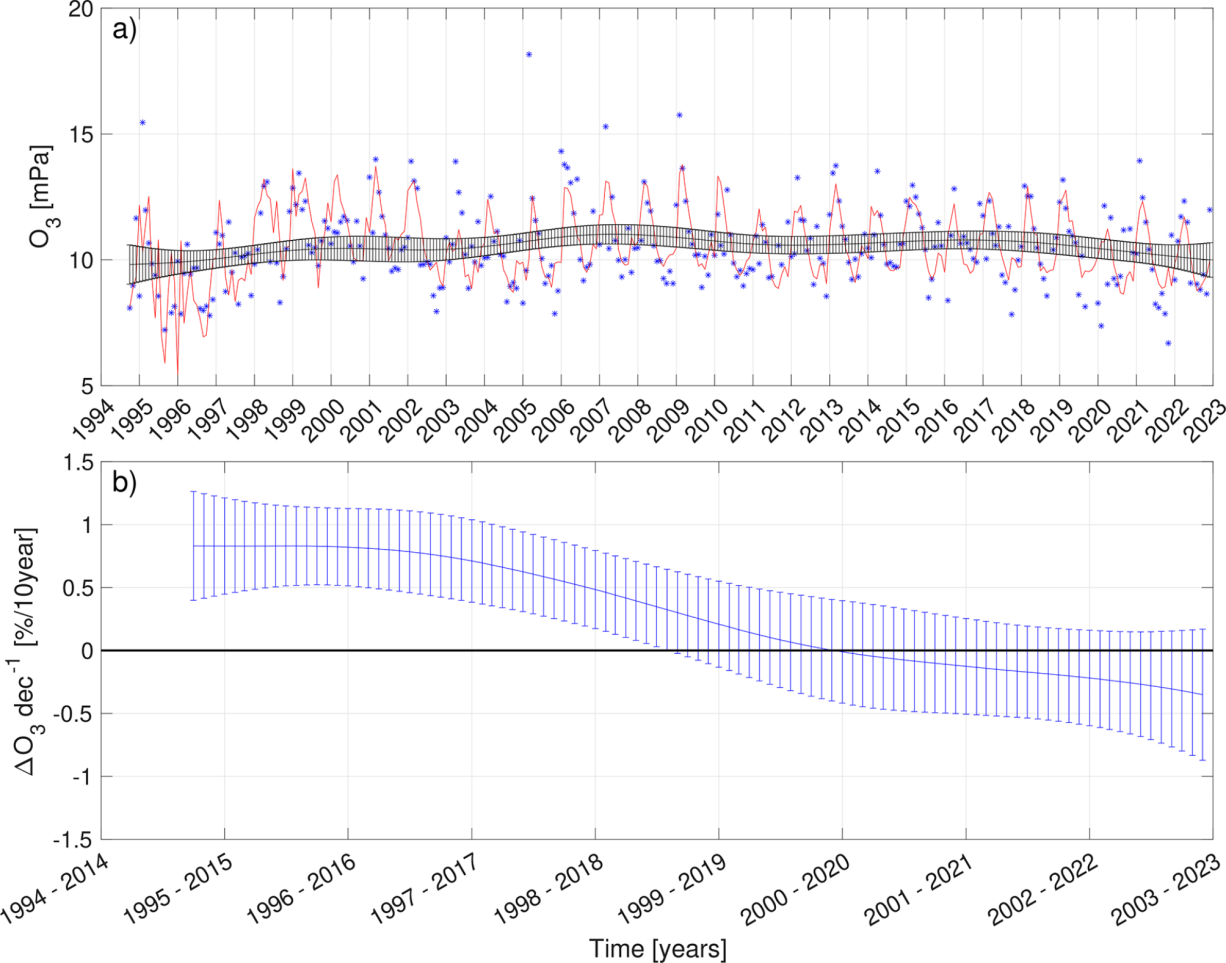
The modeled result of the time series from Sodankylä at $\textrm{L}_{4}$ altitude layer. In panel (**a**), the blue dots are monthly and height averaged ozone time series. The red line following the dots is the DLM fit. The black line is the local level component from the DLM fit, with a 95% confidence interval as error bars. In panel (**b**), time-varying trends over 20-year periods, with a 95% confidence interval as error bars.

**Figure 2:**
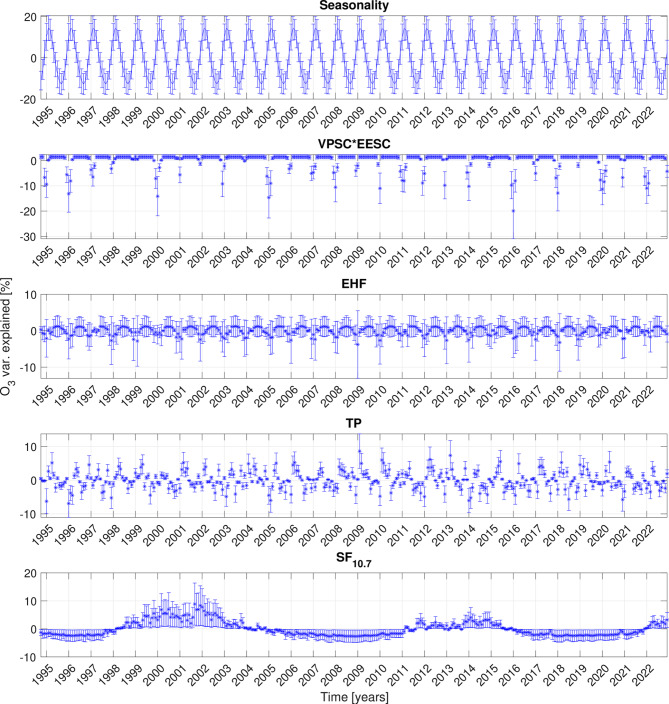
Variation explained by the DLM components that are fitted to the time series from Sodankylä at $\textrm{L}_{4}$ altitude layer. The mean value and 95% confidence intervals of the seasonality (top panel) and the proxy components (below top panel). The seasonality is the sum of the two non-conjugate terms. The proxies are their respective regression coefficient multiplied by the proxy variable. The units of the components are calculated into [%] by dividing by the mean of the ozone time series and multiplying by 100.

Figures 1 and 2 show a demonstration of the modeled results to the $\mathrm {L_4}$ altitude ozone concentration time series at the Sodankylä station. Figure 1a displays the DLM (red line) fitted to the observed (blue dots) monthly and height-averaged ozone time series (in $\mathrm {[mPa]}$). From the DLM, we have the local level component (black line) that represents the background level of the time series. Note that in the DLM framework, this component can evolve slowly and smoothly over time (see Methods). Figure 1b presents the time-varying trends over 20-year periods (in $[\%\mathrm {dec^{-1}}]$). This is a 20-year prior difference of the local level component at each time step, i.e., each month. As an example, the trends over 20-year period ending in 2015 are positive and significant. This indicates that the background level of the time series has increased significantly from 1995 to 2015. This can also be visually inspected in Fig. 1a by comparing the local level component between the years 1995 to 2015.

Figure 2 illustrates the different components (seasonal cycle and external forcers) in the DLM fit to the $\mathrm {L_4}$ layer ozone concentrations over Sodankylä (percentages are obtained by dividing by the mean of the observed ozone partial pressure). Note, that the regression coefficients for the proxies are static, while dynamic for the seasonality (see Methods). To summarize the variation explained by the fitted components from the DLM, we calculate the range, which is the sum of the absolute minimum and maximum values. The range of variation explained by seasonality, VPSC*EESC, TP and SF is 27%, 21%, 16% and 11%, respectively. The EHF is 5%, but not significant at the 95% confidence level for this time series.

**Figure 3:**
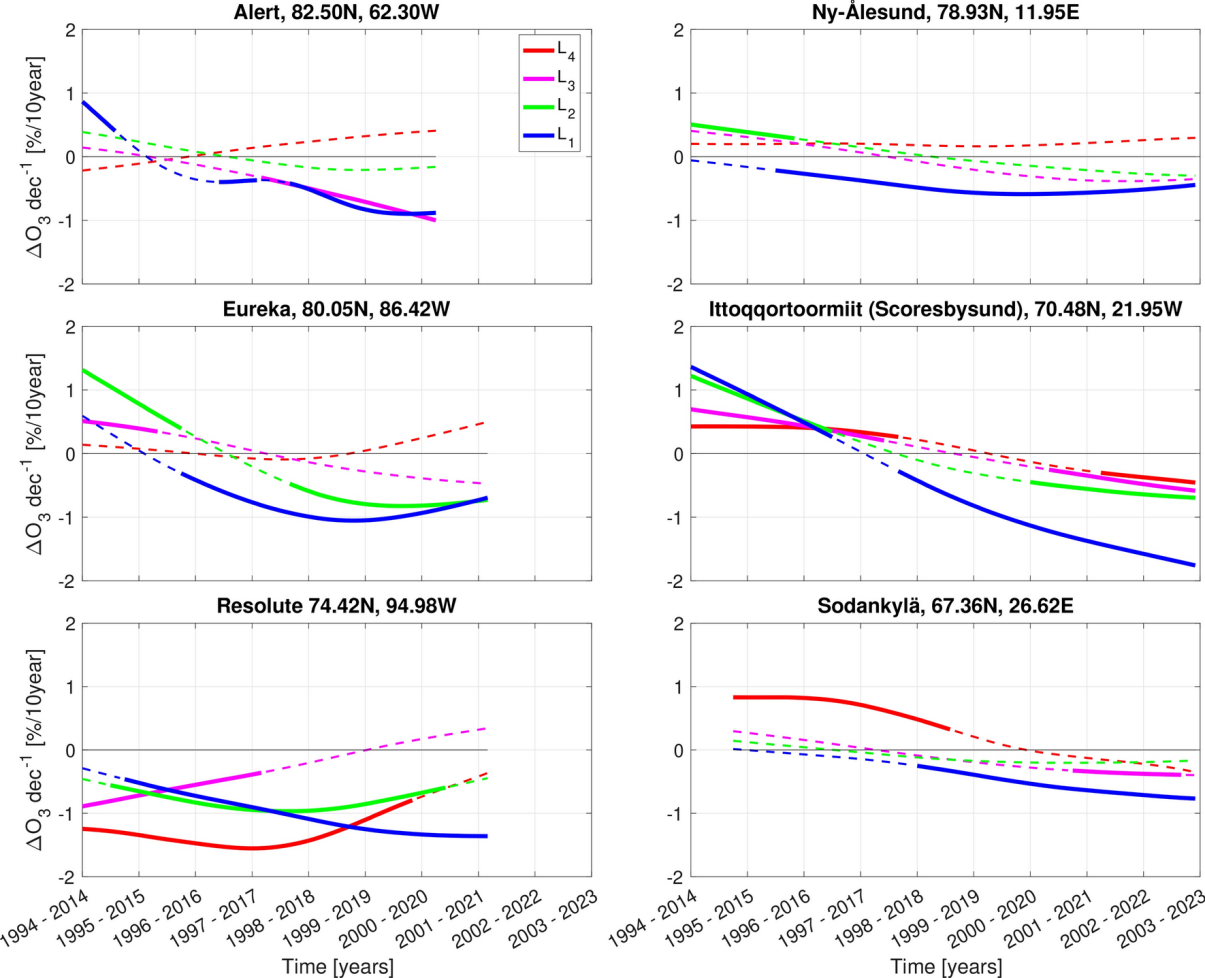
Time-varying trends over 20-year periods in the time series from all 6 stations and at all four altitude layers. Thick solid lines indicates 95% statistically significant trends while thin dashed lines indicates trends that are not significant at the 95% confidence level. The altitude layers are: $\mathrm {L_1~surface-tropopause}$; $\mathrm {L_2~tropopause-150~hPa}$; $\mathrm {L_3~150-40~hPa}$; $\mathrm {L_4~40-15~hPa}$.

Figure 3 presents the time-varying ozone trends over 20-year periods (in $[\% \mathrm {dec^{-1}}]$) in the time series from all 6 Arctic ozonesonde stations and at all altitude layers (see Fig. S2 in supplement for the corresponding local levels from all time series). At altitudes within the lower stratosphere ($\mathrm {L_2}$–$\mathrm {L_4}$) we generally find statistically significant positive trends over 20-year periods ending in 2017 or before, varying between 0.23$\pm 0.20$ and 1.31$\pm 0.76$% per decade. In contrast, we find significant negative trends above Resolute in this time period that varies between − 0.37$\pm 0.35$ and − 1.55$\pm 0.66$% per decade. Furthermore, for all sites (except Ny-Ålesund), we find rather statistically significant negative trends over 20-year periods ending in 2019 or later, with magnitudes ranging from − 0.30 ± 0.25 to − 1.00 ±0.85% per decade.

In the troposphere ($\mathrm {L_1}$), statistically significant positive trends over 20-year periods ending in 2015 and 2017, and before, are seen above Alert and Ittoqqortoormiit, respectively, with values that vary between 0.30$\pm 0.24$ and 1.36$\pm 0.45$% per decade. In the following years, we observe only significant negative trends with magnitudes varies between − 0.31$\pm 0.27$ and − 1.76$\pm 0.41$% per decade at all considered stations.

In Table 2 we show a summary of the variation explained by the DLM components that are fitted to the time series. In general, the amount of variation explained by the seasonality and the TP components are largest at the $\mathrm {L_2}$ altitude layer and decreases with higher altitude and in the troposphere $\mathrm {L_1}$. The variation explained by seasonality is smallest at the $\mathrm {L_1}$ and $\mathrm {L_4}$ altitude layers. The AO is significant at the 95% confidence level for all stations, except Sodankylä. The range of the variation in the ozone time series explained by the AO for Alert, Eureka and Resolute is a factor of roughly 1.6 times greater than for Ny-Ålesund and Ittoqqortoormiit. The VPSC*EESC contribution is significant only for the $\mathrm {L_4}$ altitude layer. We also note that this component is significant at all stations, except Resolute. The range of variation explained by the VPSC*EESC is largest for Alert and Ittoqqortoormiit, moderate for Ny-Ålesund and Sodankylä, and lowest for Eureka and Resolute. The EHF and SF components are considerably less significant than the other proxies. However, they still contribute to the model’s performance by reducing the residual trend that remains.

The bottom 4 rows of Table 2 show the adjusted $\mathrm {R^2}$, i.e., the estimated total variation explained by the model. We note that this number is low for some of the time series. However, the model diagnostics (Figs. S4 to S27 in the supplementary file), show that the residuals are normally distributed and un-correlated. This means that the modeled results captures the underlying trend in the time series. The low $\mathrm {R^2}$ values indicates that the data is noisy and may provide large standard deviation for the estimated regression coefficients.

## Discussion

At the altitudes covering the lower stratosphere ($\mathrm {L_2}$–$\mathrm {L_4}$) we find statistically significant positive trends over 20-year periods ending in 2017 or before, above Eureka, Ny-Ålesund, Ittoqqortoormiit and Sodankylä. However, significant negative trends are seen above Resolute during the same time period, which highlights regional differences in our results. Sofieva et al.^30^ report trends in polar stratospheric ozone for the period 2003-2018. They find positive trends above Europe and Greenland, and negative trend above Resolute (see Fig. 10 at 25 km altitude in Sofieva et al.^30^), which are similar to our results at $\mathrm {L_4}$ altitude layer. The mechanism behind this regional difference is not clear, but Arosio et al.^32^ suggests it may be related to zonal differences in the Brewer-Dobson circulation^33,34^. Additionally, Karpetchko et al.^35^ have shown that the Arctic vortex is on average shifted toward Eurasia and suggests this can lead to regional differences in long-term ozone trends as well. Resolute is located southward compared to other Canadian stations. Therefore the observations over Resolute are expected to be less affected by the Arctic Vortex.

Ozone recovery is foreseen, due to the decline in ODS. However, the Arctic ozone recovery is expected to be relatively slow because of changes in the Arctic vortex and an observed long-term tendency towards an increase in PSC formation potential^15^. Difficulties detecting a significant increase in Arctic stratospheric ozone have been previously reported by Weber et al.^24^. Large inter-annual variability in the Arctic stratosphere is also expected to complicate detection of expected ozone recovery^4^.

In our trend model we have taken into account changes in both VPSC and EESC. Transport processes are included via seasonality, tropopause pressure and eddy heat flux. The model captures the underlying trend of the time series and explains up to 81% of the total variability observed in the stratosphere. Nevertheless, a large majority of the trends over 20-year periods ending in 2019 or later we find to be negative (up to 1% per decade), and about half of those are statistically significant at the 95% confidence level. Several of the trends over 20-year periods are positive, but non-significant. We find a trend in solar flux over the two solar cycles, but this does not explain the observed small negative trends in the stratosphere (See Fig. S3).

While a null result might be expected, as noted above, the resulting significant negative trends over 20-year periods ending in 2019 or later are interesting, given the expectation of ozone recovery, and suggest long-term changes in the Arctic vortex that are not explained by the known forcings in the regression, which are considered in our trend model. Further long-term monitoring of Arctic ozone levels may finally provide evidence of the ozone recovery.

Our results from the troposphere ($\mathrm {L_1}$) show significant positive trends over 20-year periods ending in 2015 and 2017, or before, above Alert and Ittoqqortoormiit, respectively. In the years thereafter, we find only significant negative trends above all stations. Law et al.^20^ reports observed monthly vertical trends in tropospheric ozone, at altitudes between 925 to 400 hPa, for the periods 1993–2013 and 1999–2019, using the same stations as we have in our study (See Fig. S8 in the supplement by Law et al.^20^). They generally found positive winter trends in 1993–2013 above all stations, except above Resolute, which shows negative trend. However, in the period 1999–2019 they generally found negative trends. These findings are generally in-line with our results. Law et al.^20^ discuss that the more frequent positive phases of the AO in recent years may be contributing to the observed negative trends. However, in contrast to Law et al., we included the AO as a proxy for the tropospheric ozone in our trend model. Although the AO significantly contributes in our trend model to explain the variability, a negative trend remains for the most recent 20-year periods. This means that the AO cannot fully explain those negative trends and further investigation is needed to identify the cause.

## Summary

After the peak in stratospheric levels of Ozone Depleting Substance (ODS) was reached around the year 2000, long-term trend studies on polar ozone have focused on monitoring for recovery. Robust evidence of recovery in ozone has already been found above Antarctica. The Arctic ozone, on the other hand, has a substantially higher inter-annual variation, which poses challenges for long-term trend studies aiming to detect evidence of change.

The Dynamic Linear Model (DLM) is based on a hierarchical Bayesian approach and state space framework that allows the model to derive trends with non-linear features, which can be expected in atmospheric ozone time series. Here, we have used a DLM to detect time-varying trends over 20-year periods in time series of vertical ozone (in [mPa]), measured in-situ by ozonesondes from 6 Arctic stations, in the time period 1994–2022. The data were prepared into monthly and height averaged time series, using the altitude layers: surface-tropopause ($\mathrm {L_1}$), tropopause—150 hPa ($\mathrm {L_2}$), 150–40 hPa ($\mathrm {L_3}$) and 40–15 hPa ($\mathrm {L_4}$). The model takes into account the effect of seasonality, external forcers by proxies and $\mathrm {1^{st}}$-order correlation by adding noise. For the external forcers, we have included process-oriented proxies for Arctic ozone, i.e., tropopause pressure (Arctic Oscillation for $\mathrm {L_1}$), volume of polar stratospheric cloud multiplied with the effective equivalent stratospheric chlorine, eddy heat flux and solar radio flux (10.7 cm) for the 11-year solar cycle.

In the troposphere ($\mathrm {L_1}$), we find significant positive trends over 20-year periods ending in 2015 and 2017, or before, above Alert and Ittoqqortoormiit, respectively, which vary between 0.30±0.24 and 1.36 ±0.45% per decade. In the following years, we find only significant negative trends over 20-year periods at all stations, varying between − 0.31 ± 0.27 and − 1.76 ± 0.41% per decade. At altitudes within the lower stratosphere ($\mathrm {L_2}-\mathrm {L_4}$), we find a regional difference in the trends over 20-year periods ending in 2017 or before. Significant positive trends, varying between 0.23 ± 0.20 and 1.31 ± 0.76% per decade, are found above Eureka, Ny-Ålesund, Ittoqqortoormiit and Sodankylä. However, in contrast, significant negative trends, varying between − 0.37 ± 0.35 and − 1.55 ± 0.66% per decade are found above Resolute during the same time period.Then the trends over 20-year periods ending in 2019 or later in the lower Arctic stratosphere are either not statistically significant or if significant they are rather negative, varying between − 0.30 ± 0.25 and − 1.00 ± 0.85% per decade. This indicates that the recovery of Arctic lower stratospheric ozone remains undetected. Overall, this work highlights the importance of continued monitoring of Arctic ozone levels to detect evidence of ozone recovery in the future.

## Methods

### Ozonesonde and proxy data

The ozonesonde is a balloon-borne instrument, based on an Electrochemical Concentration Cell (ECC) sensor that measures in-situ vertical profiles of ozone^36^. The vertical coverage of these instruments ranges from ground level to typically up to 30 km (10 hPa) altitude. Because some characteristics of the ECC sondes have changed over time, a homogenization of the data is required to avoid biases in long-term trend studies^37,38^. This is provided by the Harmonization and Evaluation of Ground Based Instruments for Free Tropospheric Ozone Measurements^39^ (HEGIFTOM) project. From this project, we used the data available from 6 Arctic stations (see Table 1, Fig. S1), over time period 1994 to 2022.

The ozone measurements from the sondes are given in units of [mPa]. We prepared the ozonesonde data into monthly and height averaged time series, with standard error as uncertainty. The ozone data are integrated over four atmospheric layers: the troposphere ($\mathrm {L_1}$, surface to tropopause), lowermost stratosphere ($\mathrm {L_2}$, tropopause to 150 hPa), lower stratosphere ($\mathrm {L_3}$, 150 hPa to 40 hPa), and mid-stratosphere ($\mathrm {L_4}$, 40 hPa to 15 hPa). The chosen layers are chemically and dynamically coherent^37,40^.

We have rejected profiles from an altitude layer if the sounding did not measure up to the upper limit of the layer. This is carried out to prevent anomalous values in certain months of the time series because of higher sample number from the lower limit compared to the rest of the altitude layer. Table 1 shows the number of profiles that remain after rejection at each altitude layer and from all stations. Due to these rejection rules the $\mathrm {L_4}$ altitude layer has on average $\approx 30\%$ less profiles than $\mathrm {L_1}$.

To account for external forcers that affect the trends over 20-year periods in the measured ozone time series, proxy variables are included to the model. In this work, we have chosen to include process-oriented proxies for Arctic tropospheric and lower stratospheric ozone^4,37,40,41^, which are: Volume Polar Stratospheric Cloud multiplied by Effective Equatorial Stratospheric Chlorine (VPSC*EESC), Solar radio Flux (SF) at 10.7 cm wavelength, Eddy Heat Flux (EHF) and Tropopause Pressure (TP). However, in the $\mathrm {L_1}$ layer, we use the Arctic Oscillation (AO) instead of the TP proxy.

VPSC is a proxy for the monthly mean volume of polar stratospheric clouds. Here, we calculated the volume of air between the 370 K and 550 K potential temperature levels, where the temperature is below the formation temperature of nitric acid trihydrate, using ERA5 data^9^. We assumed a formation temperature of 194 K.

The estimates of the EESC are provided by the NASA Goddard Space Flight Center^42^. In this work, we have used a specific model configuration to obtain estimates of the EESC for the Arctic^31^. This is obtained by using a model run with the WMO scenario A1 2014, the mean age of air set to 6, the age of air spectrum width is 3, fractional release type is Laube2010^43^ and bromine scaling factor set to 50.

The SF is a proxy for the 11-year solar cycle, which is known to influence the photochemical production rate of atmospheric ozone^44^. Vigouroux et al.^45^ found this proxy to be significant for a long-term data set of ozone outside the polar region. On the other hand, Bahramvash Shams et al.^40^ concluded that the solar cycle did not significantly explain any variation in Arctic ozone time series. However, as their data set encompassed only one solar cycle, they also suggested it might be a significant contributor for longer time series. In our case, we have more than two solar cycles, thus an effect may be expected.

The EHF is the Eddy Heat Flux, which is spatially averaged at 100 hPa and over 45–75°N. This proxy describes the upward Rossby wave propagation from the troposphere to the lower stratosphere^4,40,46^. Large values of EHF indicate high wave activity, thus a weakened polar vortex^4,40,46^.

The TP is a proxy for the vertical distribution of ozone related to the surface pressure system, which is related to convergence or divergence of air^47^. When the tropopause pressure is low (higher tropopause height), the lower stratospheric ozone content is lower, and higher if pressure is high^48^ (lower tropopause height).

The AO is a proxy for the large-scale meteorology that modulates the stratospheric injection of ozone into the troposphere, as well as long-range transport of ozone and its precursors, in the Arctic^49^.

### Dynamic linear model

The standard method to detect trend(s) in long-term time series is the simple or Multi-Linear Regression (MLR) analysis. However, the linear regression models assume the trends in the time series to be linear. Atmospheric composition time series, such as of ozone, are typically not time stationary^27,30^, i.e. they may have non-linear features and abrupt changes. In some cases, the non-linear features or the abrupt changes in the time series are clearly seen and may be taken into account by using a piece-wise approach of the MLR^27^. However, because the piece-wise approach requires inflection points to be inserted manually by the user, this method may provide biased statistical inference of the trend in the time series^28^.

In this study, we have used a DLM to detect 20-year trends in the ozone time series^27^. This model is based on a state space framework and hierarchical Bayesian approach, which allows the model to estimate time-varying regression coefficients whose dynamical evolution is determined by the time series. This allows the regression coefficients in the DLM to change according to the non-linear features and abrupt changes in the time series as they occur. Laine et al.^27^ compared the DLM against MLR on stratospheric ozone time series outside the polar cap regions and reported cases with significant differences in the trend, where the DLM explained a higher portion of the variation, and concluded the MLR misses important features in those cases. An additional advantage is that the Bayesian approach of the DLM allows it to better handle data gaps and errors^27,38,50^, which are present in our work with ozonesonde measurements^38^. Finally, the DLM can take the auto-regressive component into account simultaneously, and not by a separate iteration^27^. For more details and further justification for using DLM to detect long-term trends in atmospheric or climate time series, see Durbin and Koopman^51^ and Laine et al.^29^.

The Matlab Toolbox for DLM analysis, including a general description with examples is publicly available here: https://mjlaine.github.io/dlm/dlmtut.html. In this work, we have used the same implementation of the DLM as used and explained in detail by Laine et al.^27^. Here, we provide a brief description necessary to reproduce our results.

A general DLM, based on a linear state space approach with Gaussian errors, can be written as an observation (Eq. 1), and a state evolution (Eq. 2) equations

1 $$\begin{aligned} y_t&= F_tx_t+v_t,&v_t \sim N(0,V_t) \end{aligned}$$

2 $$\begin{aligned} x_t&= G_tx_{t-1}+w_t,&w_t \sim N(0,W_t) \end{aligned}$$

where $y_t$ are the observations, that is, the time series from the ozonesondes, and the *x*_*t*_ is the state of the system at time *t*. The matrices $G_t$ and $F_t$ are the state evolution operators that allow the components within $x_t$ to evolve dynamically over time, and the observation operators that transform the $x_t$ into the $y_t$, respectively. $V_t$ and $W_t$ are the covariance matrices of the errors in $y_t$ and $x_t$, respectively, which consist of the uncertainties $v_t$ and $w_t$, that are assumed to be Gaussian.

To describe the variation in the ozone time series, we implement four main components into the model: Trend, seasonality, external forcing by proxy variables and noise with auto-regressive component. These components are built into the matrices $\textrm{G}$, $\textrm{F}$ and $\textrm{W}$ in Eqs. 1 and 2. The trend is modeled as a random walk with two hidden states: The local level and the change in local level from $\textrm{t}$ to $\mathrm {t+1}$, i.e. local trend. The elements in the matrices are

3 $$\begin{aligned} G_{trend} = \begin{bmatrix} 1 & 1 \\ 0 & 1 \\ \end{bmatrix} , F_{trend} = \begin{bmatrix} 1 & 0 \\ \end{bmatrix} , W_{trend} = \begin{bmatrix} 0 & 0 \\ 0 & \sigma ^2_{trend} \end{bmatrix} \end{aligned}$$

The seasonality is modeled with an annual and semi-annual term. Including their respective conjugate terms, we obtain four hidden states. The elements are

4 $$\begin{aligned} G_{sea} = \begin{bmatrix} \cos (\pi /6) & \sin (\pi /6) & 0 & 0\\ -\sin (\pi /6) & \cos (\pi /6) & 0 & 0\\ 0 & 0 & \cos (\pi /3) & \sin (\pi /3)\\ 0 & 0 & -\sin (\pi /3) & \cos (\pi /3) \end{bmatrix} , F_{sea} = \begin{bmatrix} 1&0&1&0 \end{bmatrix} , W_{sea} = \begin{bmatrix} \sigma _{sea}^2 & 0 & 0 & 0 \\ 0 & \sigma _{sea}^2 & 0 & 0 \\ 0 & 0 & \sigma _{sea}^2 & 0 \\ 0 & 0 & 0 & \sigma _{sea}^2 \\ \end{bmatrix} \end{aligned}$$

The external forcers are modeled as a hidden state for each proxy variable. In this work, we use four proxies, so the following components are included to the system

5 $$\begin{aligned} G_{proxy} = \begin{bmatrix} 1 & 0 & 0 & 0\\ 0 & 1 & 0 & 0\\ 0 & 0 & 1 & 0\\ 0 & 0 & 0 & 1 \end{bmatrix} , F_{proxy} = \begin{bmatrix} P_{1,t}&P_{2,t}&P_{3,t}&P_{4,t} \end{bmatrix} , W_{proxy} = \begin{bmatrix} \sigma _{proxy}^2 & 0 & 0 & 0 \\ 0 & \sigma _{proxy}^2 & 0 & 0 \\ 0 & 0 & \sigma _{proxy}^2 & 0 \\ 0 & 0 & 0 & \sigma _{proxy}^2 \\ \end{bmatrix} \end{aligned}$$

where $\mathrm {P_{k,t}}$ is the value of the proxy time series at time $\textrm{t}$. The auto-regressive component allows us to remove correlation in the model residuals. In this work, we use a first-order auto-regressive model. The elements are thus

6 $$\begin{aligned} G_{ar} = \begin{bmatrix} \rho \end{bmatrix} , F_{ar} = \begin{bmatrix} 1 \end{bmatrix} , W_{ar} = \begin{bmatrix} \sigma _{ar}^2 \end{bmatrix} \end{aligned}$$

Finally, if the individual observation uncertainty is known, then $\mathrm {V_t}$ is $\mathrm {\sigma ^2_{obs(t)}}$. The complete model is then built as diagonal block matrices

7 $$\begin{aligned} G =&\begin{bmatrix} G_{trend} & 0 & 0 & 0 \\ 0 & G_{sea} & 0 & 0 \\ 0 & 0 & G_{proxy} & 0 \\ 0 & 0 & 0 & G_{ar} \\ \end{bmatrix} \end{aligned}$$

8 $$\begin{aligned} F_t =&\begin{bmatrix} F_{trend} & F_{sea} & F_{proxy} & F_{ar} \\ \end{bmatrix} \end{aligned}$$

9 $$\begin{aligned} W =&\begin{bmatrix} W_{trend} & 0 & 0 & 0 \\ 0 & W_{sea} & 0 & 0 \\ 0 & 0 & W_{proxy} & 0 \\ 0 & 0 & 0 & W_{ar} \\ \end{bmatrix} \end{aligned}$$

10 $$\begin{aligned} V_t =&\begin{bmatrix} \sigma ^2_{obs(t)} \\ \end{bmatrix} \end{aligned}$$

Here, we have dropped the subscript on $\textrm{G}$ and $\textrm{W}$ as in this case they do not depend on $\textrm{t}$, i.e. they are constant.

To estimate the regression coefficients in this model, a hierarchical Bayesian approach is used. Therefore, the prior values model error covariance matrix $\textrm{W}$ must be given as input to the model. To remain systematic in this work, we have used the same prior values for all time series. These prior values are shown in Table S1. For the trend, we have selected a small value to constrain the model to provide us a smooth and slowly varying trend, which can be expected in ozone time series^27^. The dynamical changes to the seasonality components only affect their amplitudes. In this work, we use the same value for all four seasonal components, which means their amplitudes evolve equally. The scale is larger than the other dynamical components, such as the trend, to allow more variation. For the proxies, we have set the prior values to zero, which will result in a static regression coefficient. The main reason is to obtain a more simple and straightforward model. For that, we want to remain as systematic as possible. Allowing the proxies to evolve dynamically with time can make it challenging to find a systematic set of prior values that provides a model that is fitted to the time series with an acceptable behaviour in statistical and physical sense, particularly in our case as we have a total of 28 time series to tune. The local level is also set to zero, because the change in local level is taken into account by the trend component. The initial values to the 1st order auto-regressive components are also required as input. These values are different for each time series to ensure that the 1st lag in the model residuals is never significantly correlated, i.e. we obtain an acceptable model for the time series. These values are summarized in Table S2. Finally, for the error estimation for each model parameter, we used Markov Chain Monte Carlo (MCMC) simulations with 10 000 iterations.

When the specifications to fit a DLM to the observed time series are known, they are given as input to the **dlmfit** function, which is a part of the Matlab Toolbox provided by Laine et al.^27^. In the supplementary file, we show the model performance to each time series (Figs. S4–S27), that is, a residual diagnostic and distribution plot of the prior and posterior values to the DLM parameters. The residual diagnostics shows that the residuals of the modeled results to the time series are normal and independent. This means that the modeled results are consistent with the data and they capture the underlying trend. The prior and posterior distributions of $\sigma _{trend}$ show small values, which indicates that the model supports the search for slowly varying background variability in the ozone time series. The posterior distribution of the seasonality is wide and variable between the time series. This indicates that the seasonality parameters vary more between the time series, which is expected since we have set the scale to a higher value. For the $\sigma _{ar}$, the posterior distribution is relatively narrow compared to the prior, which means the auto-regressive parameters can accurately be obtained from the observed time series. Finally, and most important, the posterior distributions of $\sigma _{trend}$ and $\sigma _{AR}$ are relatively different from the priors, which indicates that the model parameters are mostly determined by the observed time series and not by the prior values. This means that the trend we obtain from the DLM fit to the observed time series is determined by the time series, i.e., the data themselves.

**Table 1:** Summary of the data from the selected Arctic stations from HEGIFTOM used in this work.

Station	Latitude	Longitude	Time period	Profiles at $\mathrm {L_1}$	Profiles at $\mathrm {L_2}$	Profiles at $\mathrm {L_3}$	Profiles at $\mathrm {L_4}$
Alert	82.50$^\circ$N	62.30$^\circ$W	01/1994–04/2020	1214	1193	996	745
Eureka	80.05$^\circ$N	86.42$^\circ$W	01/1994–03/2021	1794	1761	1590	1224
Resolute	74.42$^\circ$N	94.98$^\circ$W	01/1994–03/2021	997	959	778	558
Ny-Ålesund	78.93$^\circ$N	11.95$^\circ$E	01/1994–12/2022	2410	2385	2230	1912
Ittoqqortoormiit	70.48$^\circ$N	21.95$^\circ$W	01/1994–12/2022	1477	1465	1372	1200
Sodankylä	67.36$^\circ$N	26.62$^\circ$E	10/1994–12/2022	1440	1435	1356	1113

**Table 2:** Variation explained (in % with respect to the mean of the time series) by the DLM components that are fitted to the time series.

	Alert	Eureka	Resolute	Ny-Ålesund	Ittoqqortoormiit	Sodankylä
Seasonality						
$\mathrm {L_4}$	40*	29*	31*	29*	23*	27*
$\mathrm {L_3}$	33*	36*	40*	37*	37*	47*
$\mathrm {L_2}$	58*	60*	61*	61*	60*	65*
$\mathrm {L_1}$	22*	22*	22*	24*	26*	34*

TP (AO for $\textrm{L}_{1}$)						
$\mathrm {L_4}$	32*	39*	25*	35*	30*	16*
$\mathrm {L_3}$	38*	43*	31*	41*	46*	37*
$\mathrm {L_2}$	58*	50*	34*	49*	55*	56*
$\mathrm {L_1}$	17*	12*	19*	3*	7*	1

VPSC*EESC						
$\mathrm {L_4}$	28*	16*	14	20*	30*	21*
$\mathrm {L_3}$	1	6	7	5	1	6
$\mathrm {L_2}$	3	3	11	5	1	8
$\mathrm {L_1}$	1	3	2	3	1	2

EHF						
$\mathrm {L_4}$	26*	32*	20*	4	8	5
$\mathrm {L_3}$	9	11*	5	0	1	13*
$\mathrm {L_2}$	4	8	6	9	2	7
$\mathrm {L_1}$	10	7	13*	4	3	9

SF						
$\mathrm {L_4}$	3	1	6	5	3	11*
$\mathrm {L_3}$	7	2	18*	1	2	5
$\mathrm {L_2}$	7	5	1	2	7	4
$\mathrm {L_1}$	12*	8	2	3	3	1

Adjusted $\textrm{R}^{2}$						
$\mathrm {L_4}$	40	55	44	46	27	30
$\mathrm {L_3}$	53	75	65	73	62	67
$\mathrm {L_2}$	73	81	68	81	69	71
$\mathrm {L_1}$	17	17	12	58	29	16

The asterisk sign indicates 95% significance. The bottom row shows the adjusted $\textrm{R}^{2}$ (in %) of the complete model.

When an acceptable model is obtained, we can use the local level component to infer 20-year change(s) from the time series. However, to also account for uncertainty in the fitted model, we draw 1 000 realizations of the local level component from the posterior distribution. For each realization and at each time step of the local level component, we calculate the difference between time $\textrm{t}$ and 20-years prior. Then, we calculate the mean and standard deviation of the realizations. This provides us with a mean of time-varying trends over 20-year periods, with 95% confidence intervals, for the time series.

## Supplementary Information

Supplementary Information.

## Data Availability

The Matlab Toolbox for DLM analysis were obtained from this website http://helios.fmi.fi/~lainema/dlm/. The Arctic Oscillation, Tropopause Pressure and the Solar Flux (10.7cm) were obtained from National Oceanic and Atmospheric Administration (NOAA). The Arctic Oscillation is obtained from this website https://www.cpc.ncep.noaa.gov/products/precip/CWlink/daily_ao_index/ao.shtml. The Tropopause Pressure is obtained at this website https://downloads.psl.noaa.gov/Datasets/ncep.reanalysis.derived/tropopause/. The Solar Flux data is obtained from this website https://www.swpc.noaa.gov/products/predicted-sunspot-number-and-radio-flux. The Heat Flux data is provided by National Aeronautics and Space Administration (NASA) at this website https://acd-ext.gsfc.nasa.gov/Data_services/met/ann_data.html. The of Equivalent effective stratospheric chlorine is obtained from this website https://acd-ext.gsfc.nasa.gov/Data_services/automailer/restricted/eesc.php. The ozonesonde data are obtained from the HEGIFTOM project, which are located in this website https://hegiftom.meteo.be/datasets/ozonesondes. The VPSC data is available on a public data repository^52^ at this website https://doi.org/10.23729/2e930f19-562a-42c1-ae6f-8075b3e70761.
