# Supplementary material for: Time-varying trends from Arctic ozonesonde time series in the years 1994–2022
Source: Sci Rep. 2024 Nov 12;14:27683. doi: 10.1038/s41598-024-75364-7 (PMC11557962; doi:10.1038/s41598-024-75364-7)
Supplement: Supplementary file 1 — Supplementary Information. [file 41598_2024_75364_MOESM1_ESM.pdf]

Time-varying decadal trends from Arctic ozonesonde time series in the years 1994 to 2022: supplementary file

Estimated parameter	Prior mean	Prior Scale
σ_{level}	0	0
σ_{tr}	1/10% of O_3 obs. mean	1
σ_{sea}	1% of O_3 obs. mean	2
σ_{ar}	20% of O_3 obs. mean	1
σ_{pro}	0	0

Table S1. Prior values for the auxiliary model parameters estimated by MCMC.

	Alert	Eureka	Resolute	Ny Ålesund	Ittoqqortoormiit	Sodankylä
L4	0.20	0.20	0.05	0.30	0.10	0.10
L3	0.40	0.35	0.30	0.60	0.10	0.25
L2	0.20	0.30	0.20	0.35	0.20	0.10
L1	0.05	0.30	0.20	0.35	0.20	0.20

Table S2. Initial values for the auto-regressive component.

References

1. Pawlowicz, R. M_map: A mapping package for matlab, version 1.4m [Computer software] (last accessed: 4 april 2023) (2020). www.eoas.ubc.ca/~rich/map.html.

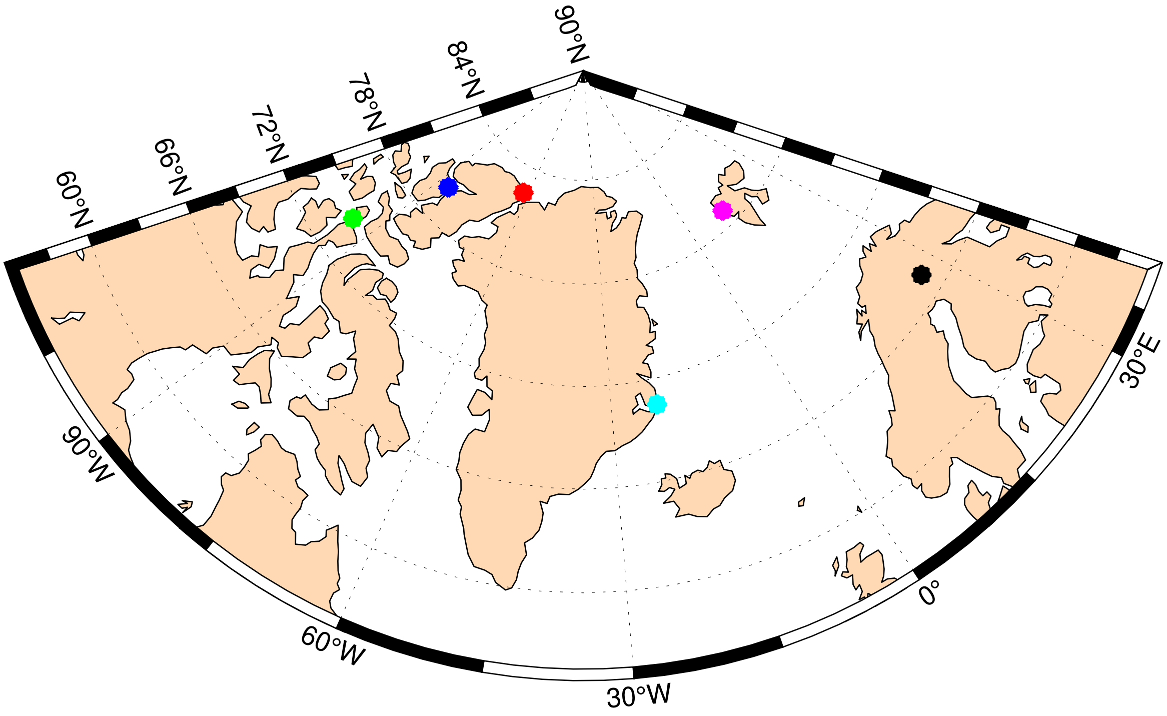

Figure S1. **Geographic locations of the Arctic ozonesonde stations.** This figure was generated by using the Matlab library M_Map[1]. The colored dots marks the stations: Alert (red), Eureka (blue), Resolute (green), Ny Ålesund (magenta), Ittoqqortoormiit (Scoresbysund, cyan) and Sodankylä (black).

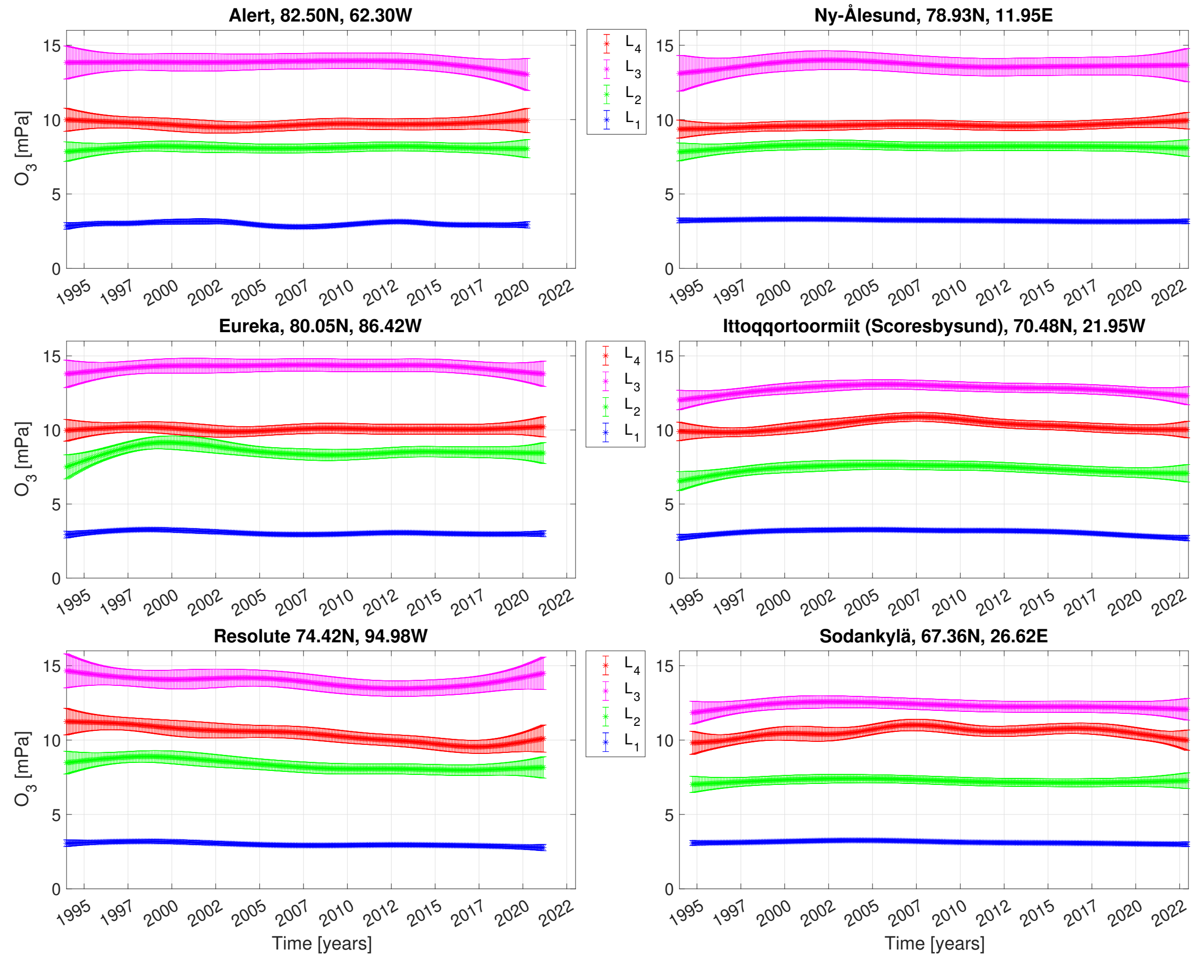

Figure S2. **Local level components from the DLM fitted to the time series.** The altitude layers are: L_1 surface − tropopause; L_2 tropopause − 150 hPa; L_3 150 − 40; L_4 40 − 15.

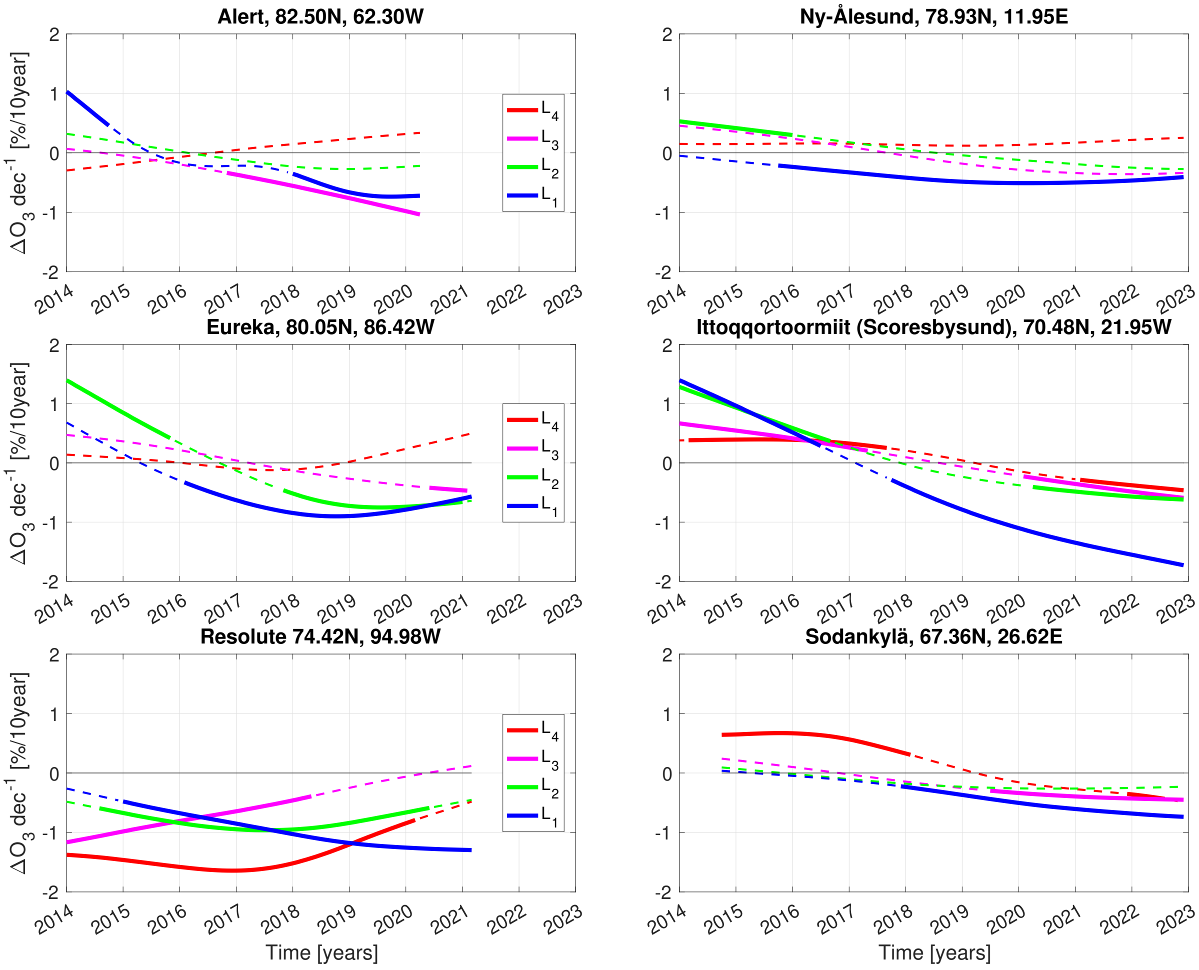

Figure S3. **Time-varying 20 year trends with de-trended solar cycle proxy.** Thick solid lines indicates 95% statistical significant while thin dashed lines indicates not significant. The altitude layers are: L_1 surface $-$ tropopause; L_2 tropopause $-$ 150 hPa; L_3 150 $-$ 40; L_4 40 $-$ 15.

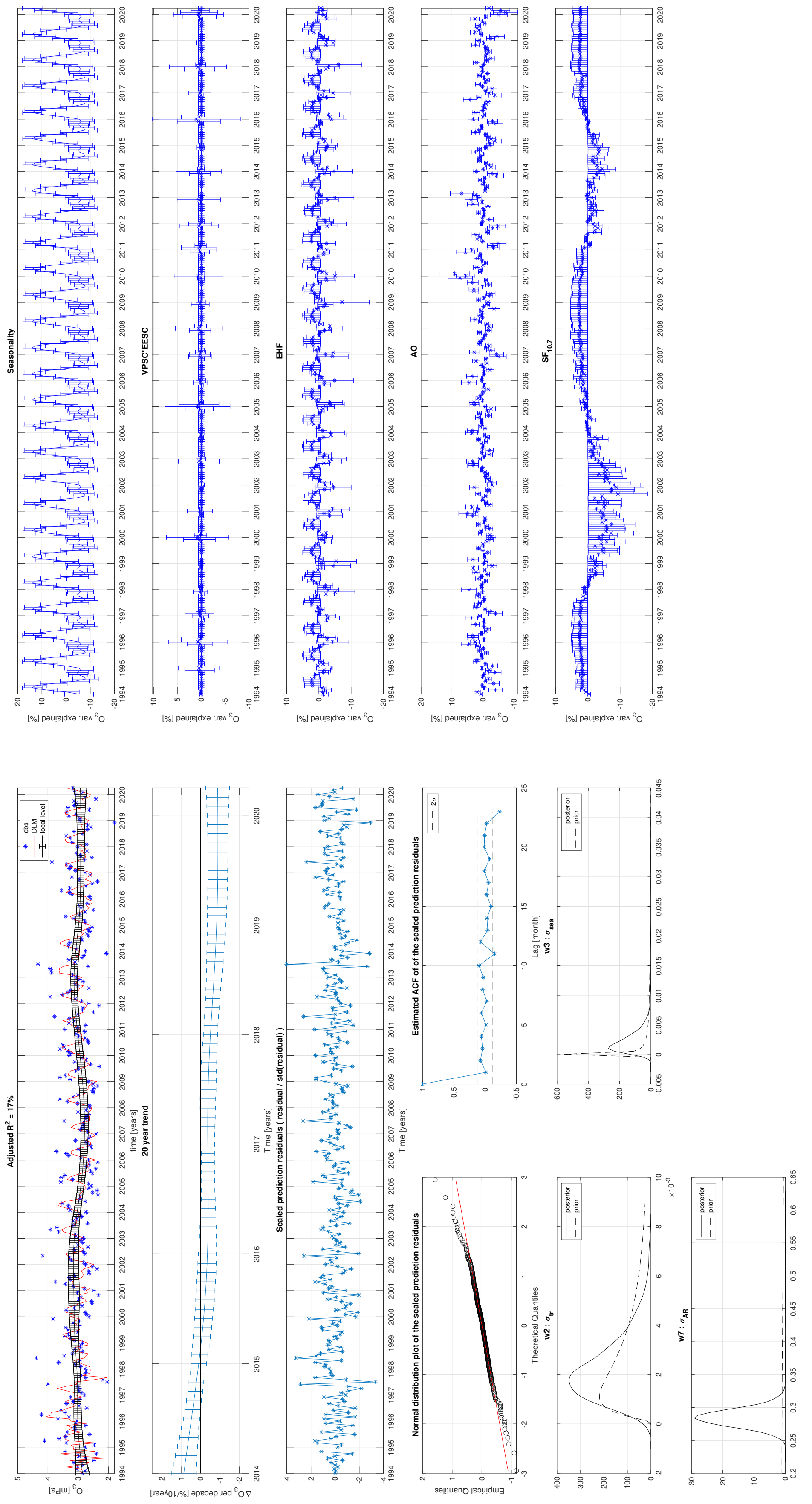

Figure S4. Alert at L_1 altitude layer.

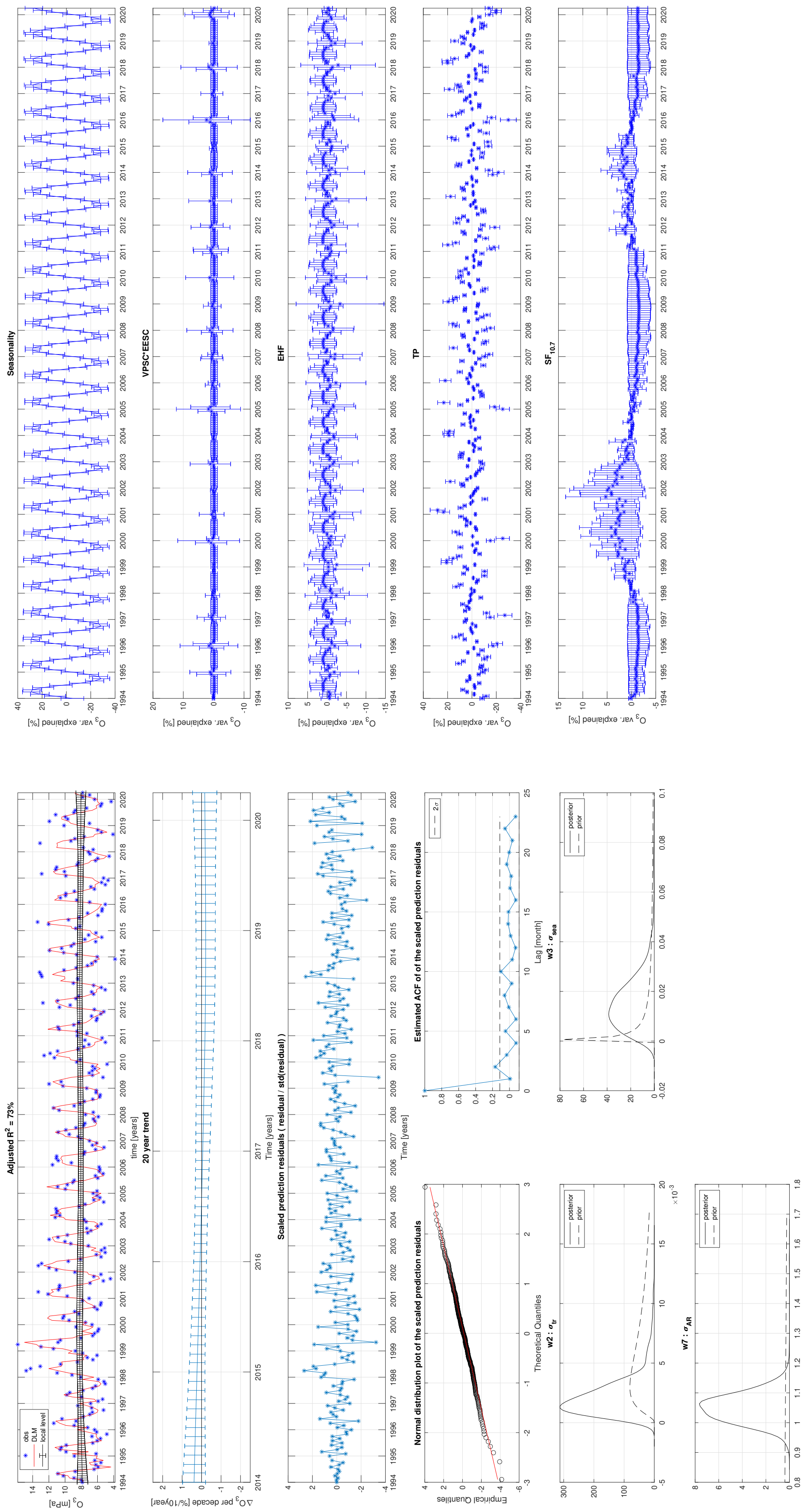

Figure S5. Alert at L_2 altitude layer.

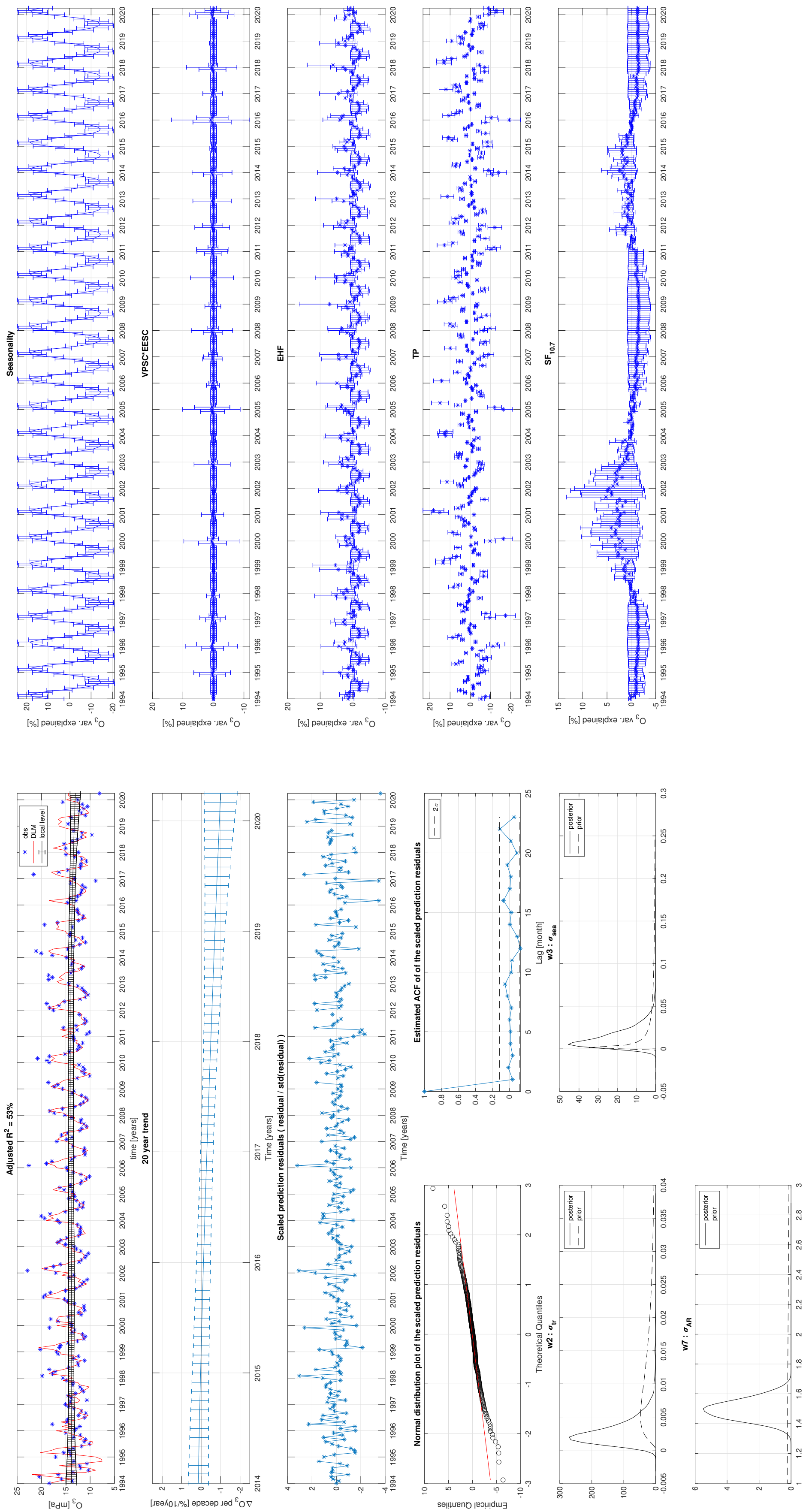

Figure S6. Alert at L_3 altitude layer.

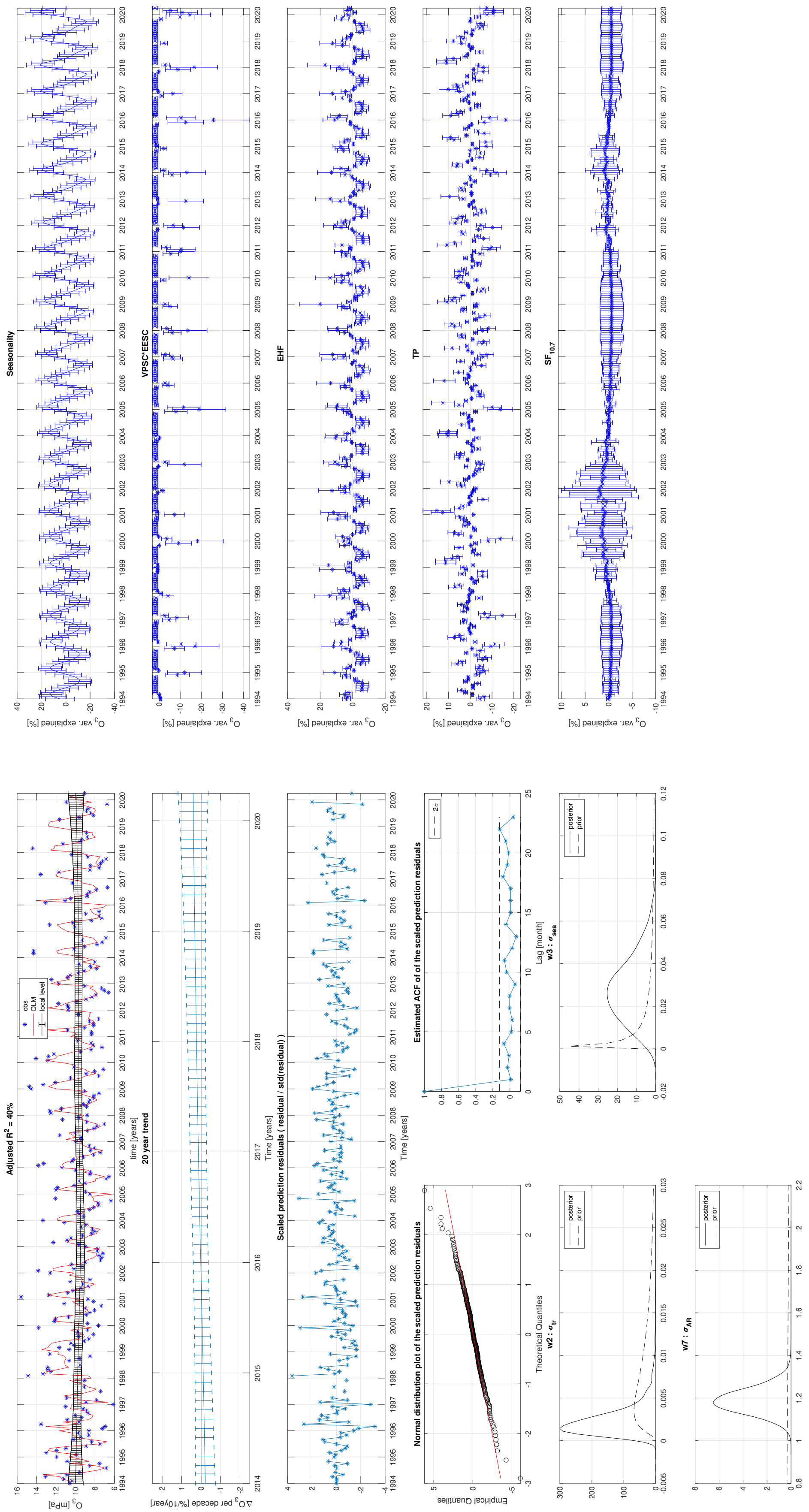

Figure S7. Alert at L_4 altitude layer.

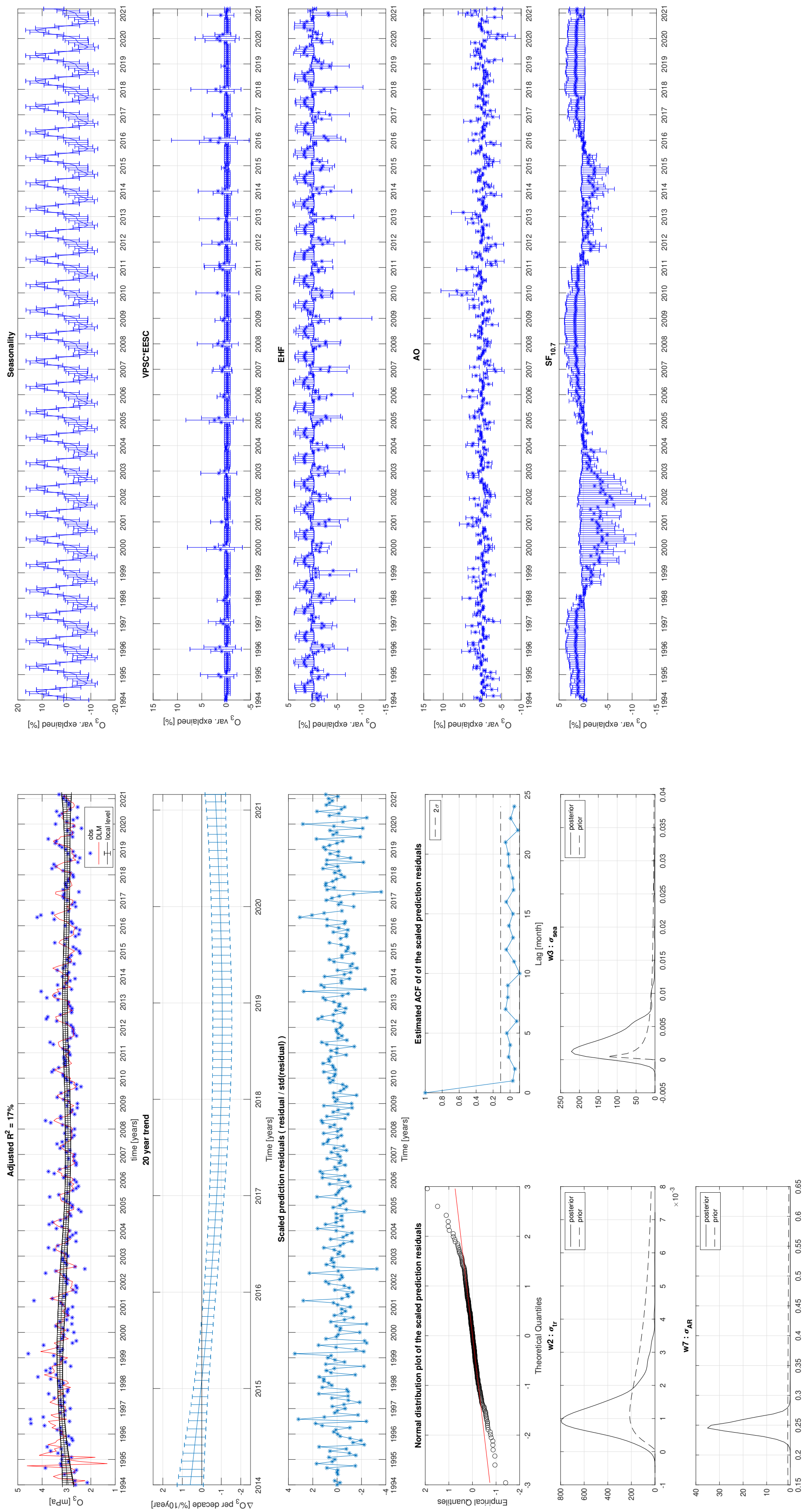

Figure S8. Eureka at L_1 altitude layer

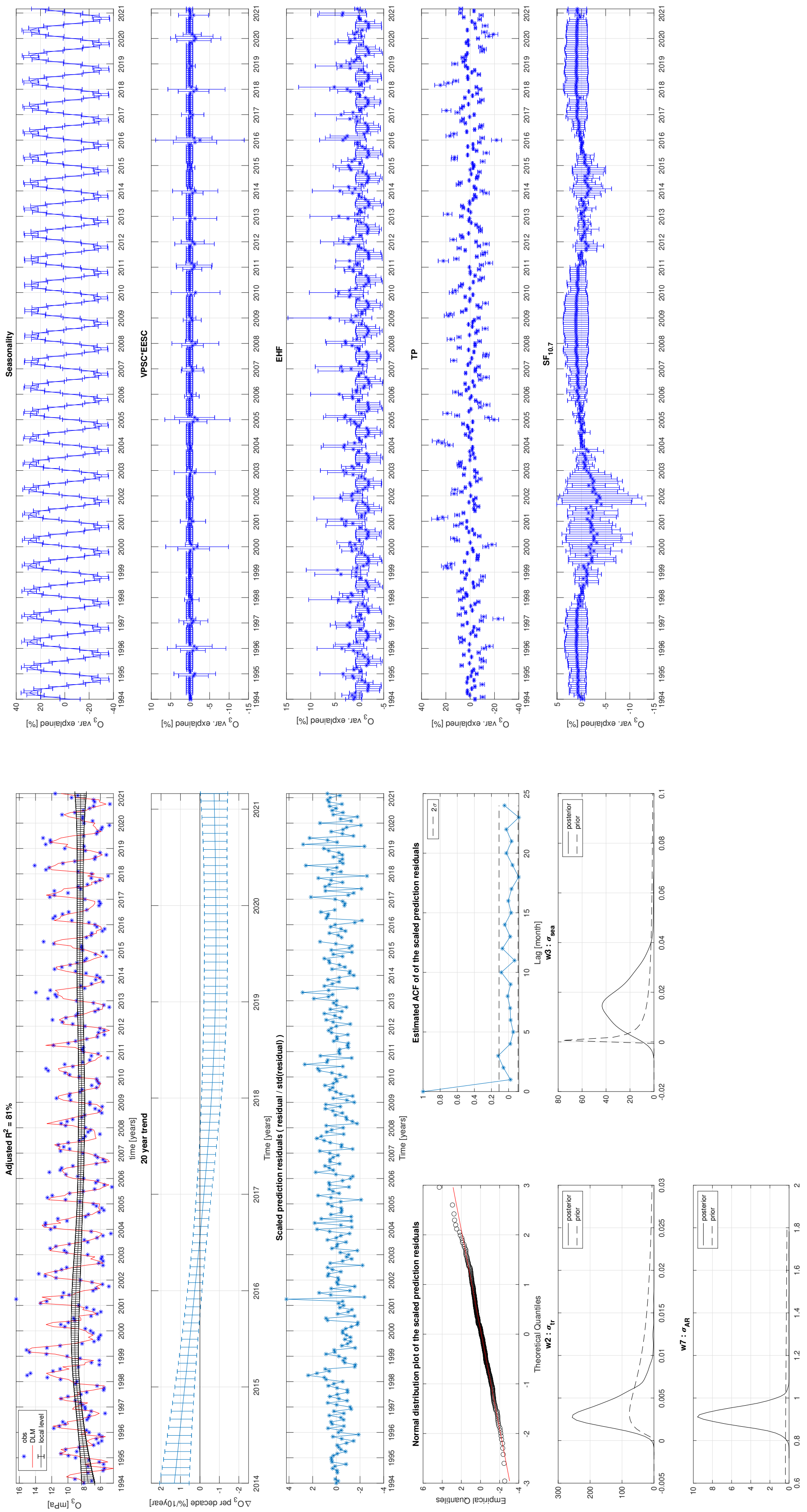

Figure S9. Eureka at L_2 altitude layer

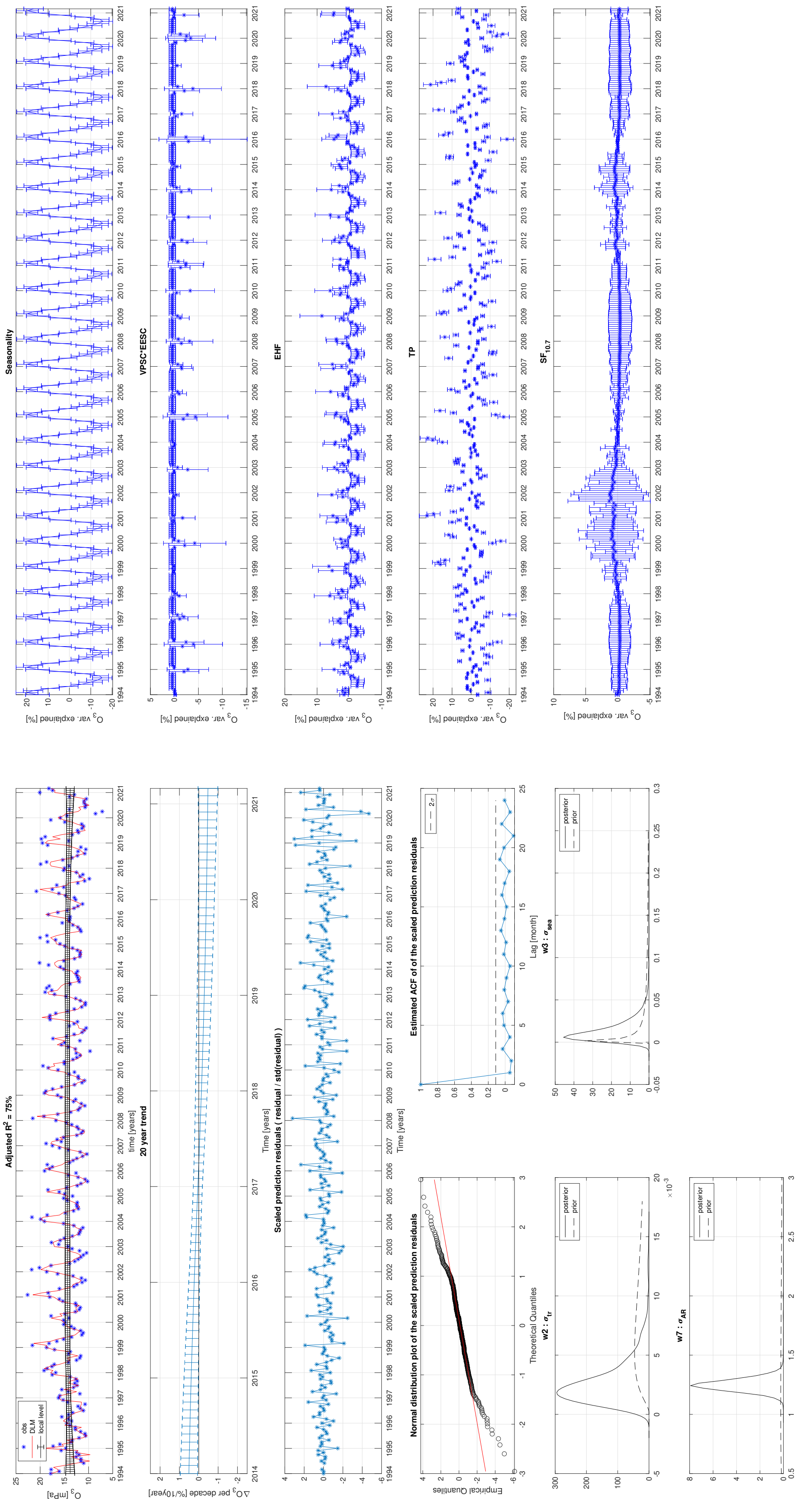

Figure S10. Eureka at L_3 altitude layer

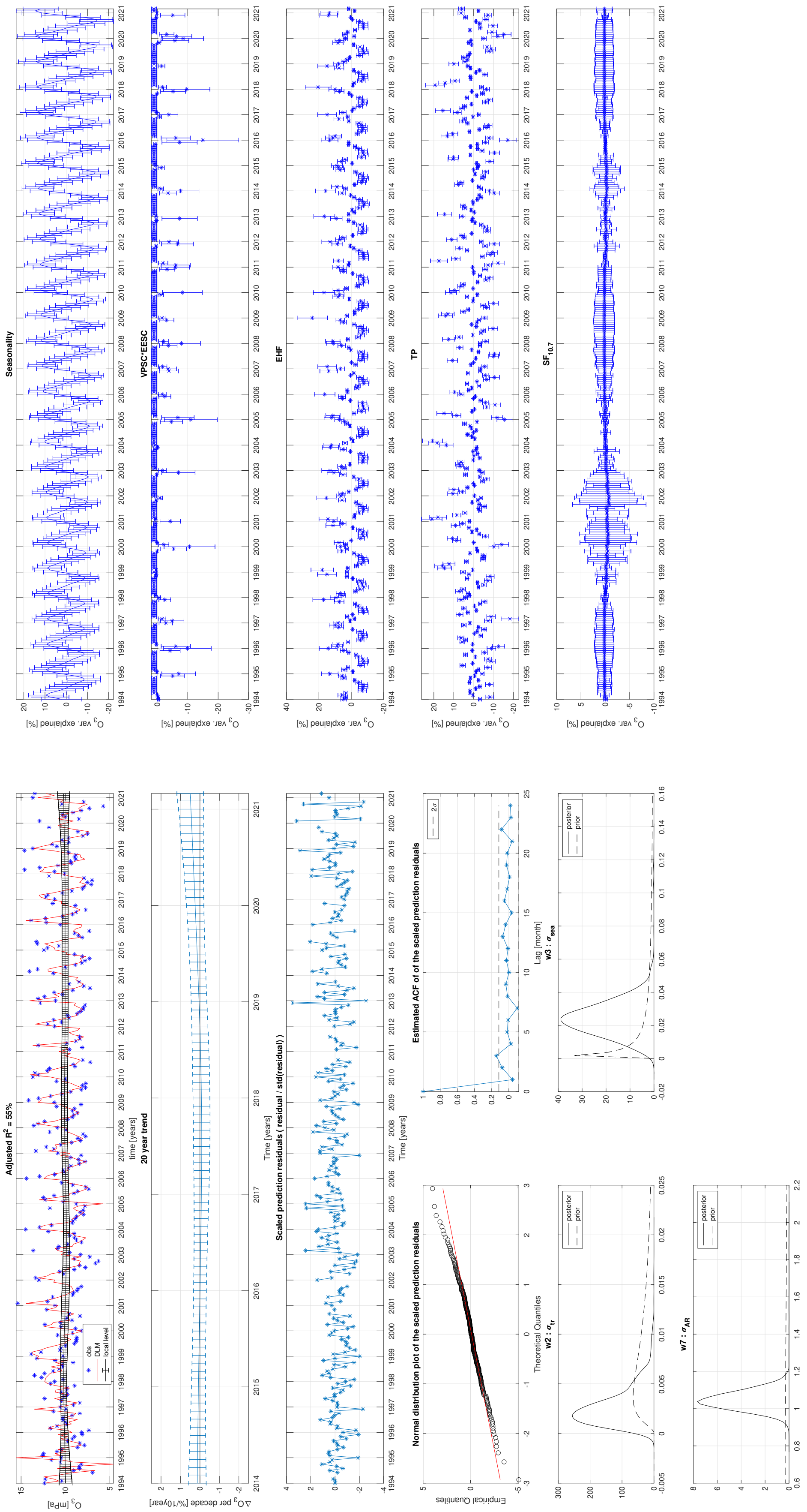

Figure S11. Eureka at L_4 altitude layer

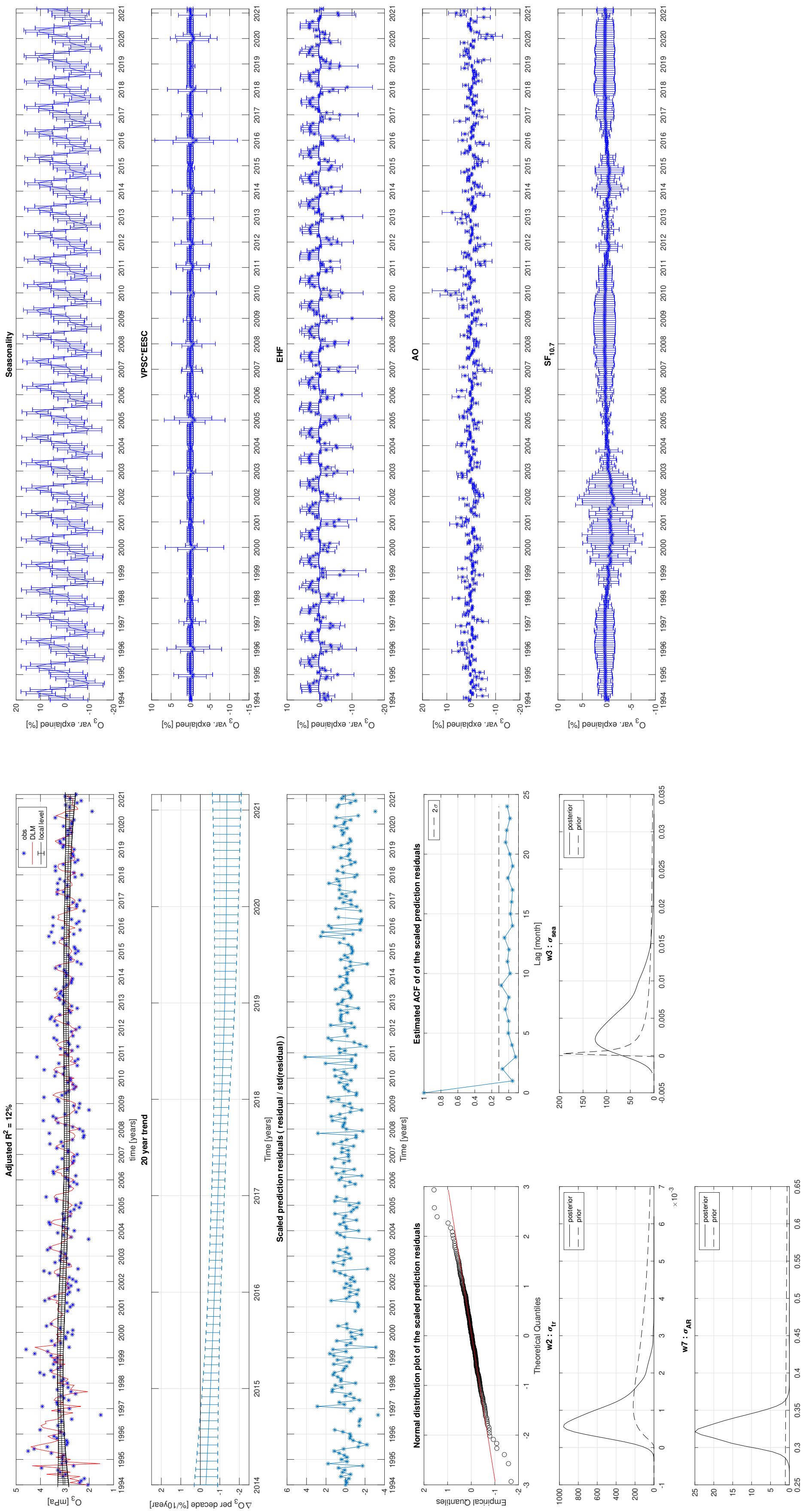

Figure S12. Resolute at L_1 altitude layer

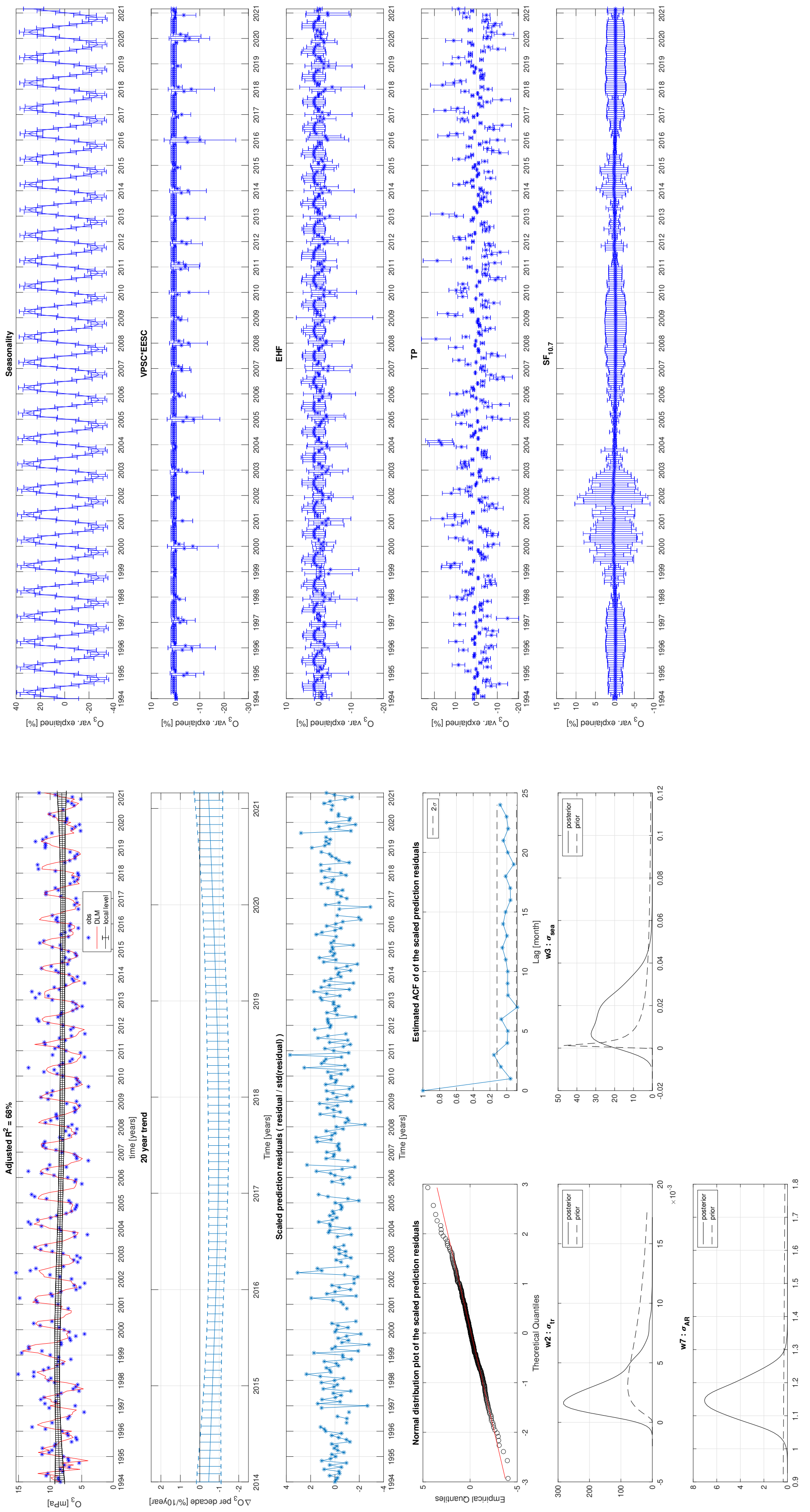

Figure S13. Resolute at L_2 altitude layer

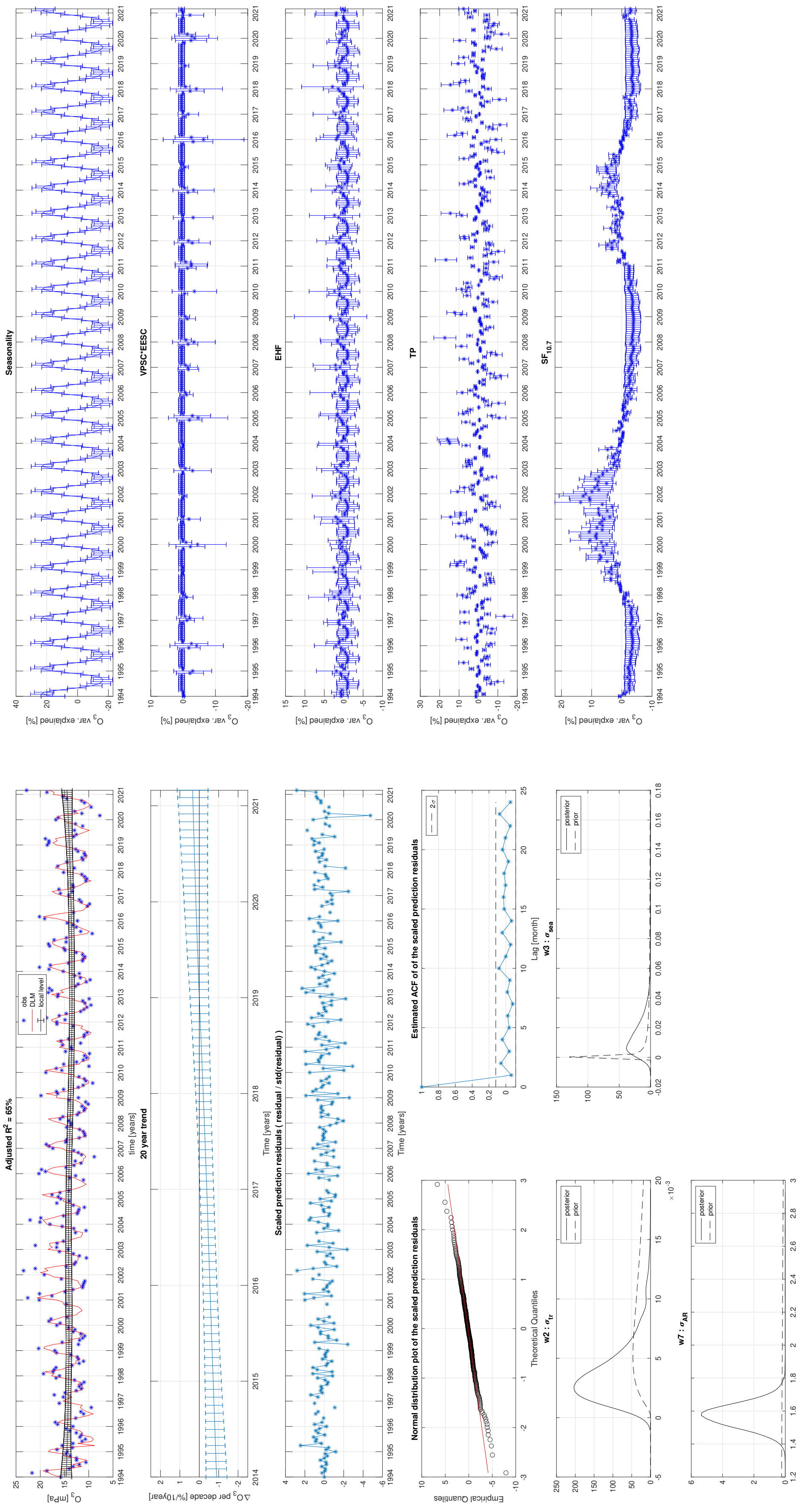

Figure S14. Resolute at L_3 altitude layer

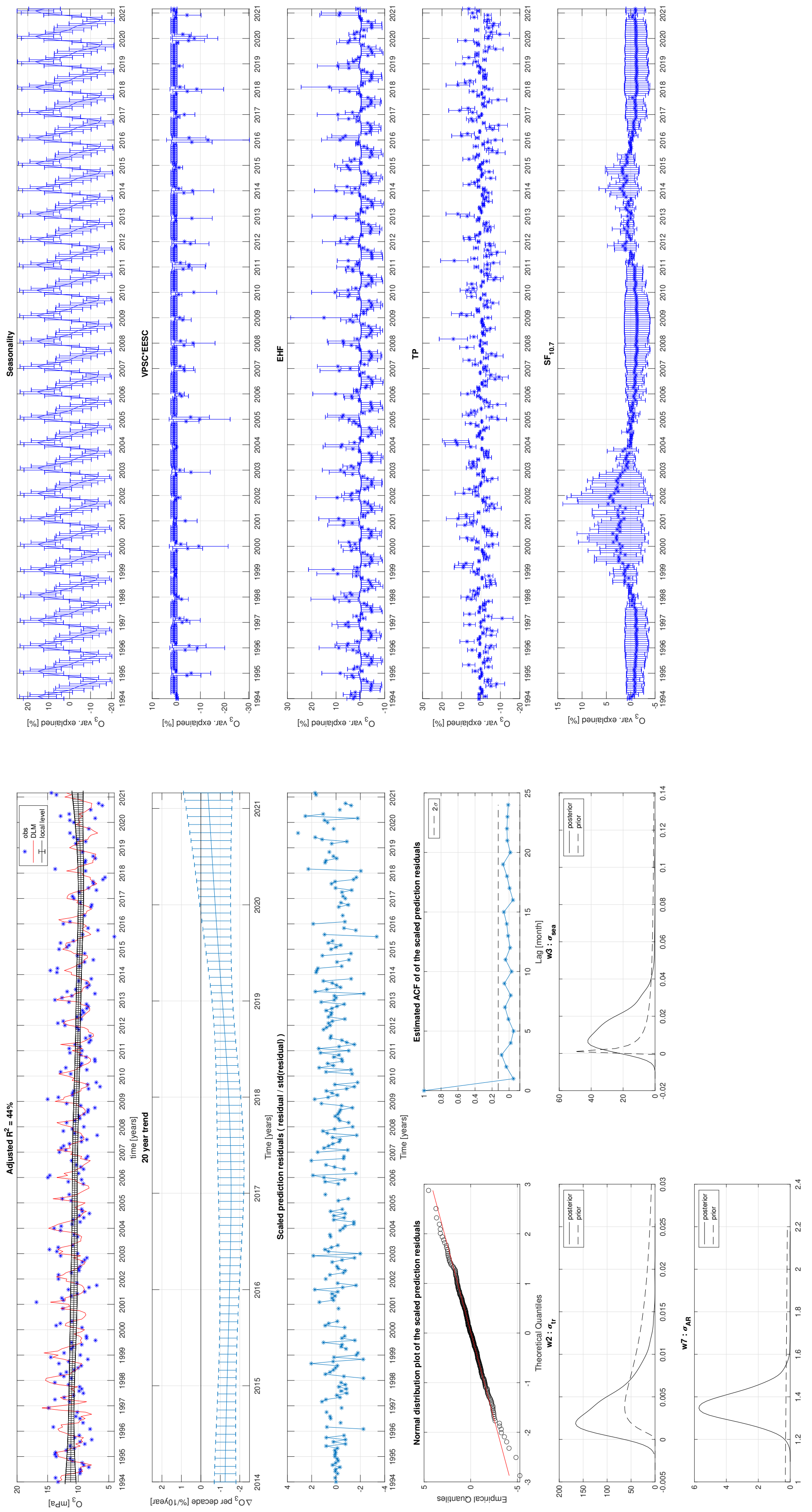

Figure S15. Resolute at L_4 altitude layer

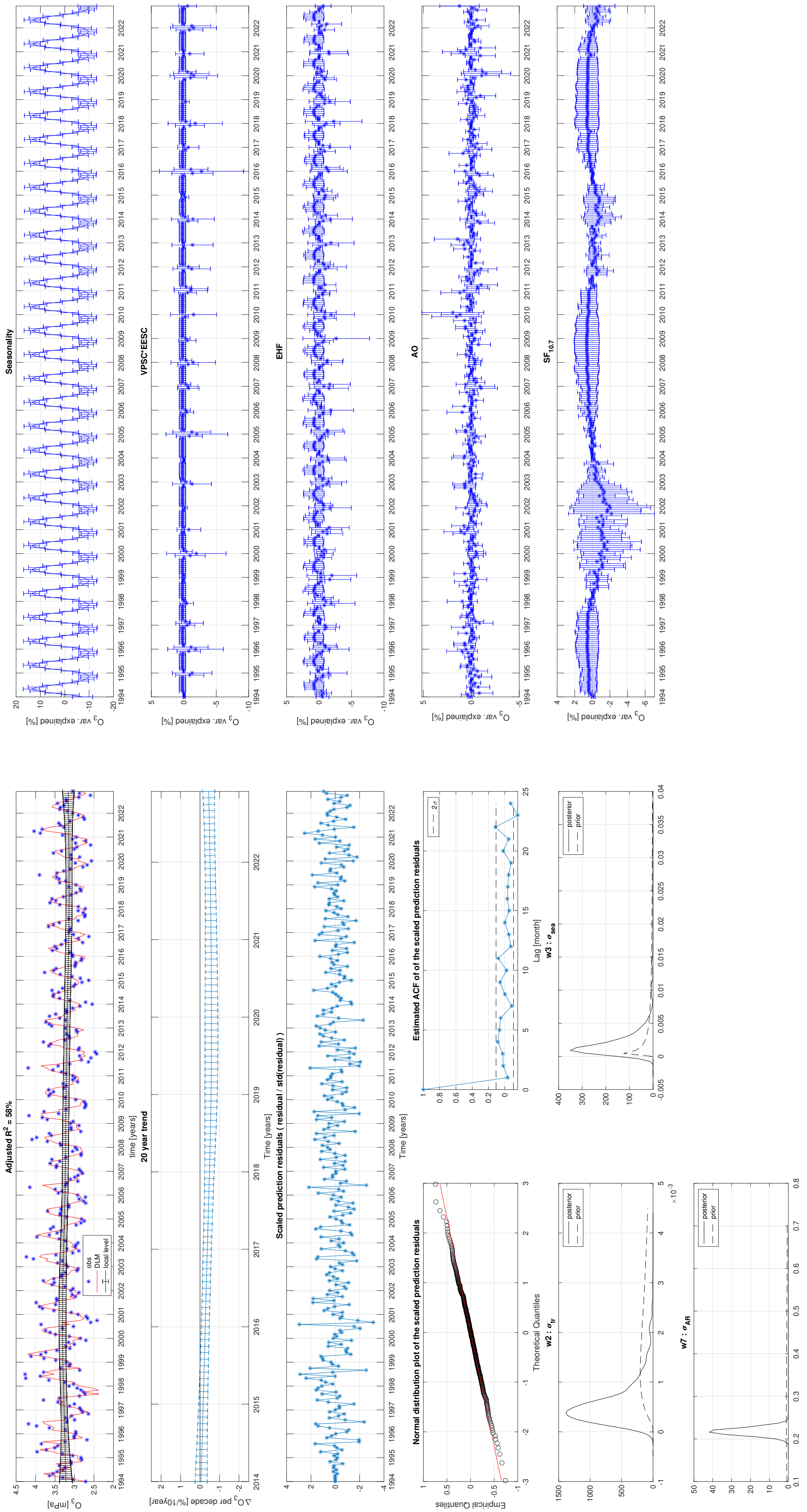

Figure S16. Ny-Ålesund at L_1 altitude layer

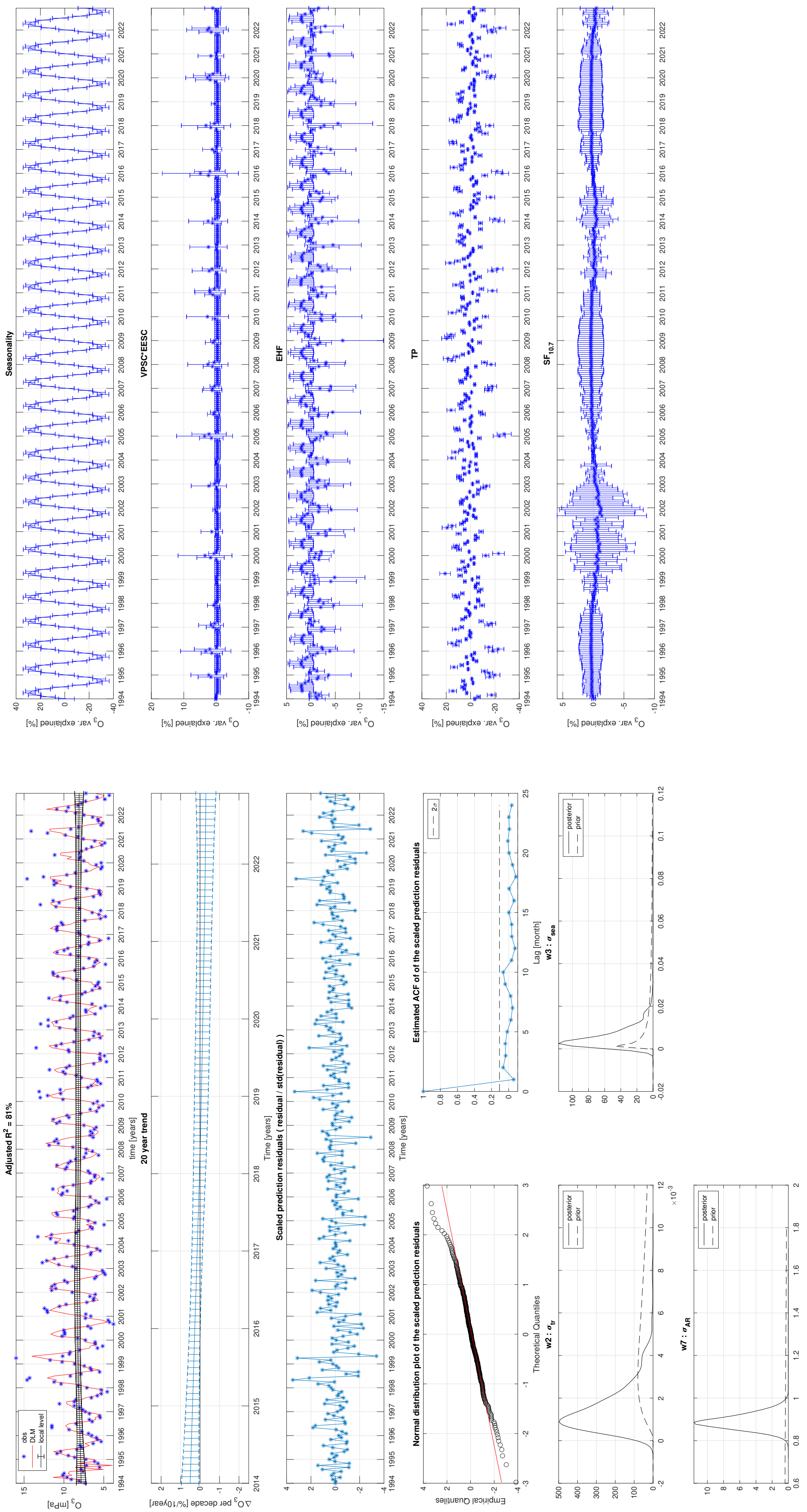

Figure S17. Ny-Ålesund at L_2 altitude layer

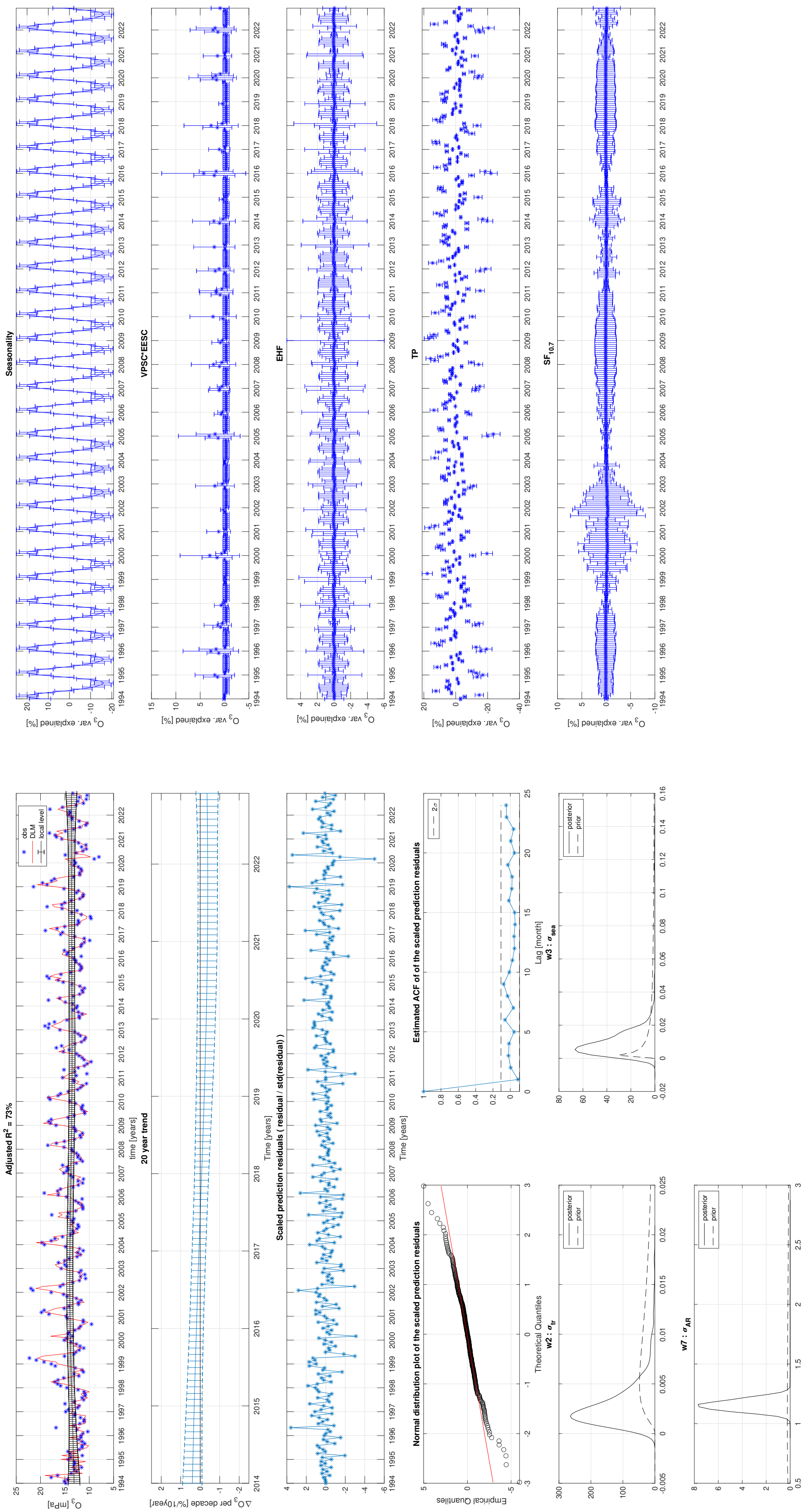

Figure S18. Ny-Ålesund at L_3 altitude layer

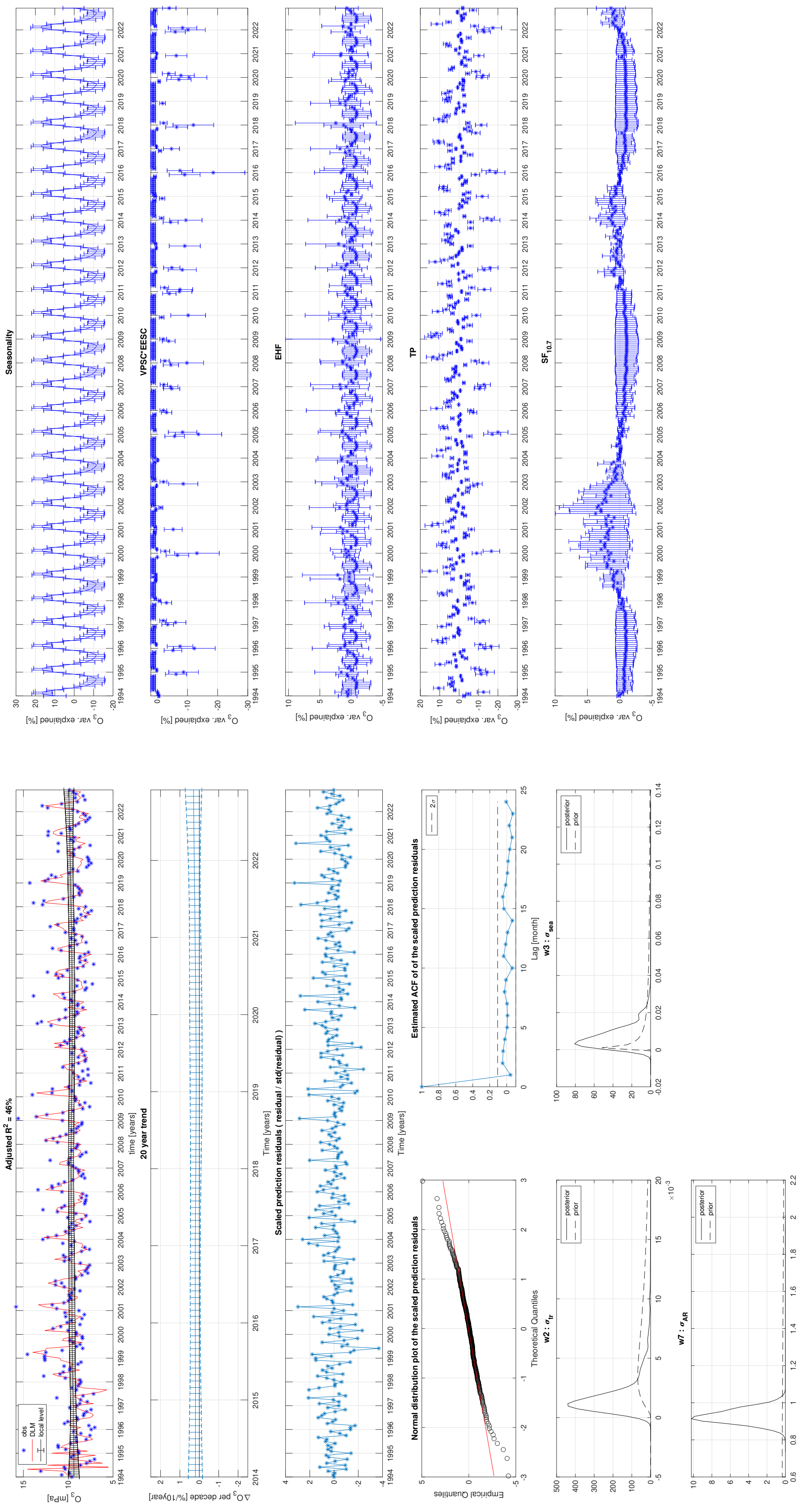

Figure S19. Ny-Ålesund at L_4 altitude layer

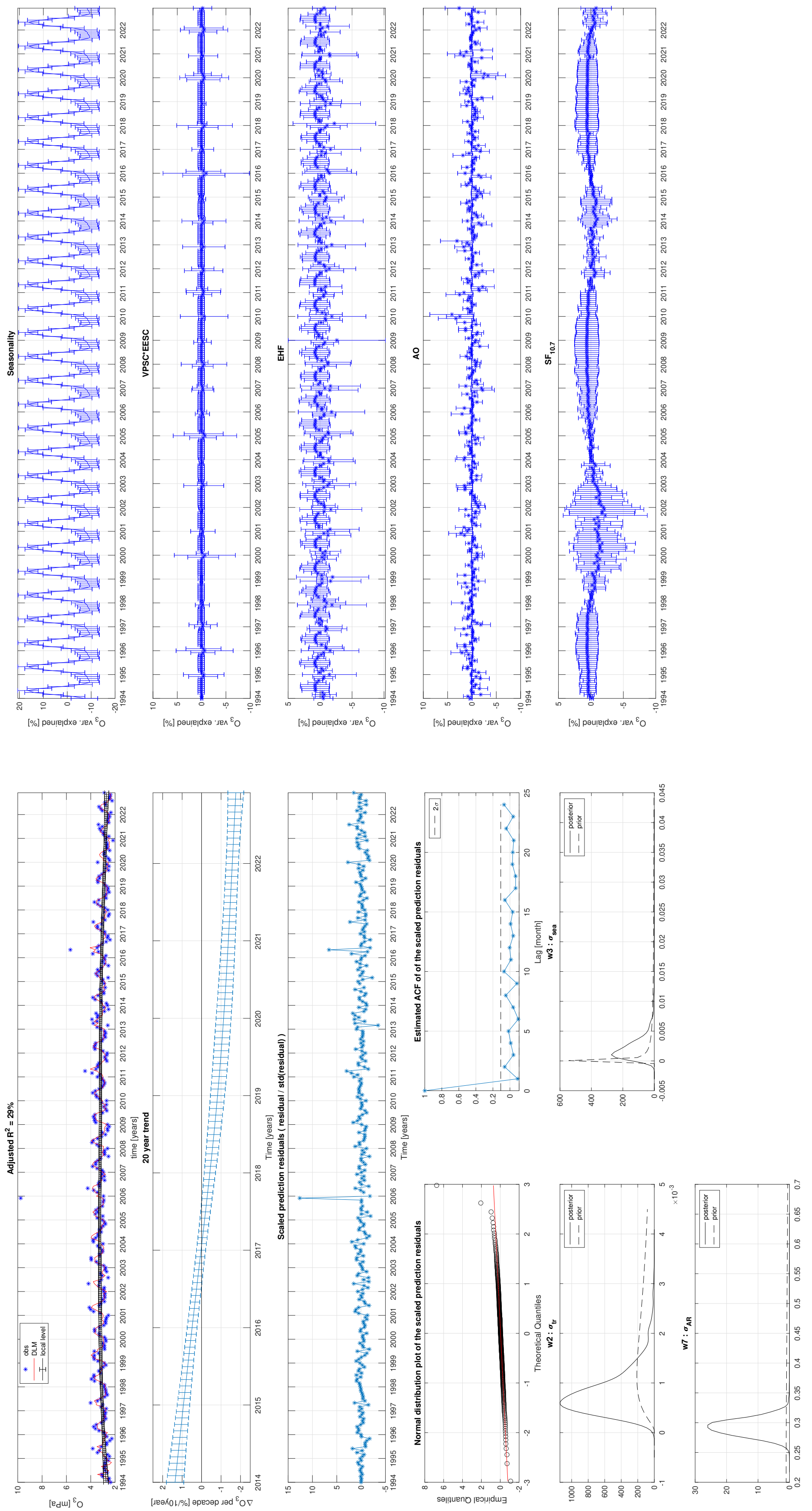

Figure S20. Ittoqqortoormiit (Scoresbysund) at L_1 altitude layer

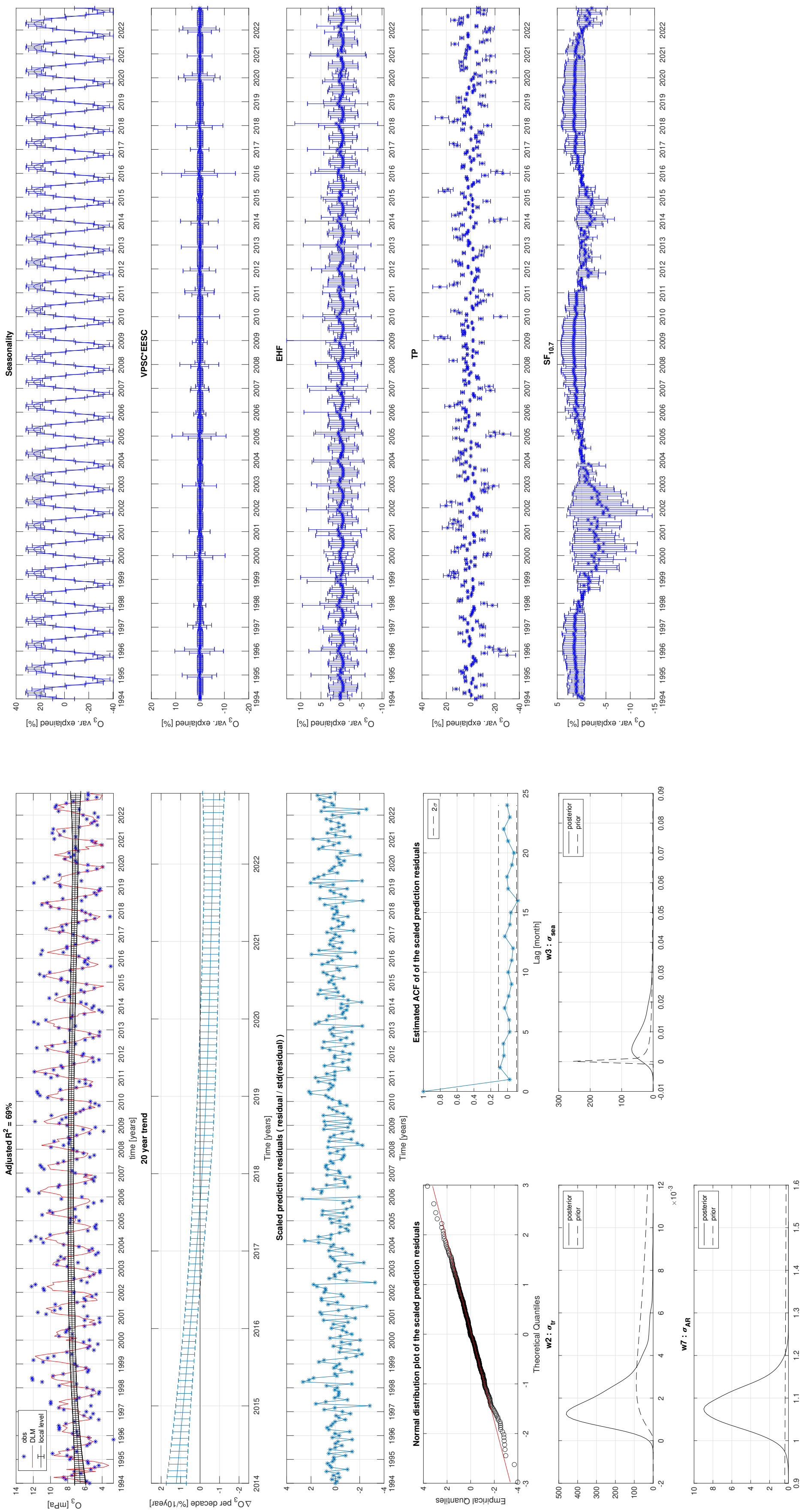

Figure S21. Ittoqqortoormiit (Scoresbysund) at L_2 altitude layer

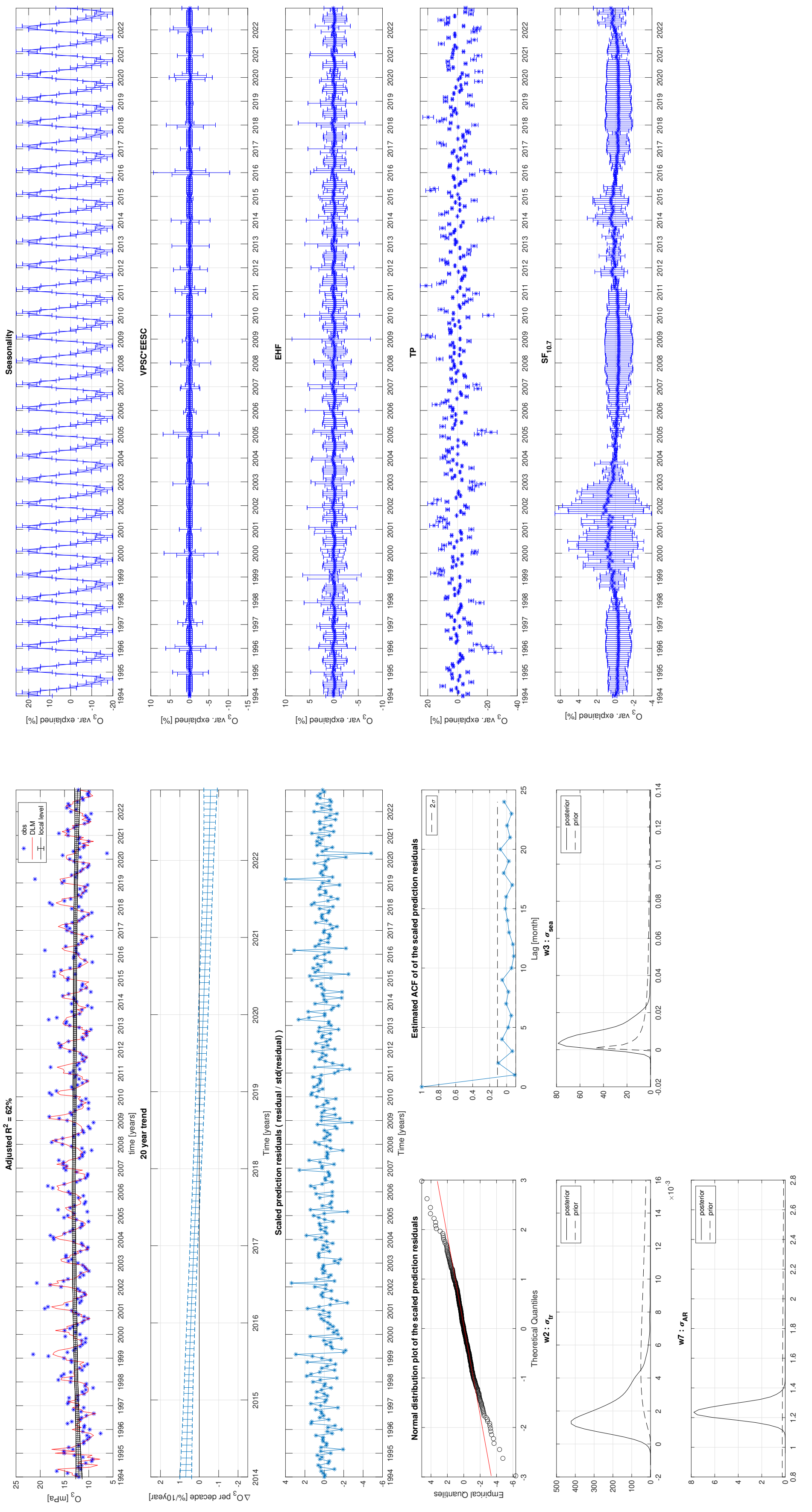

Figure S22. Ittoqqortoormiit (Scoresbysund) at L_3 altitude layer

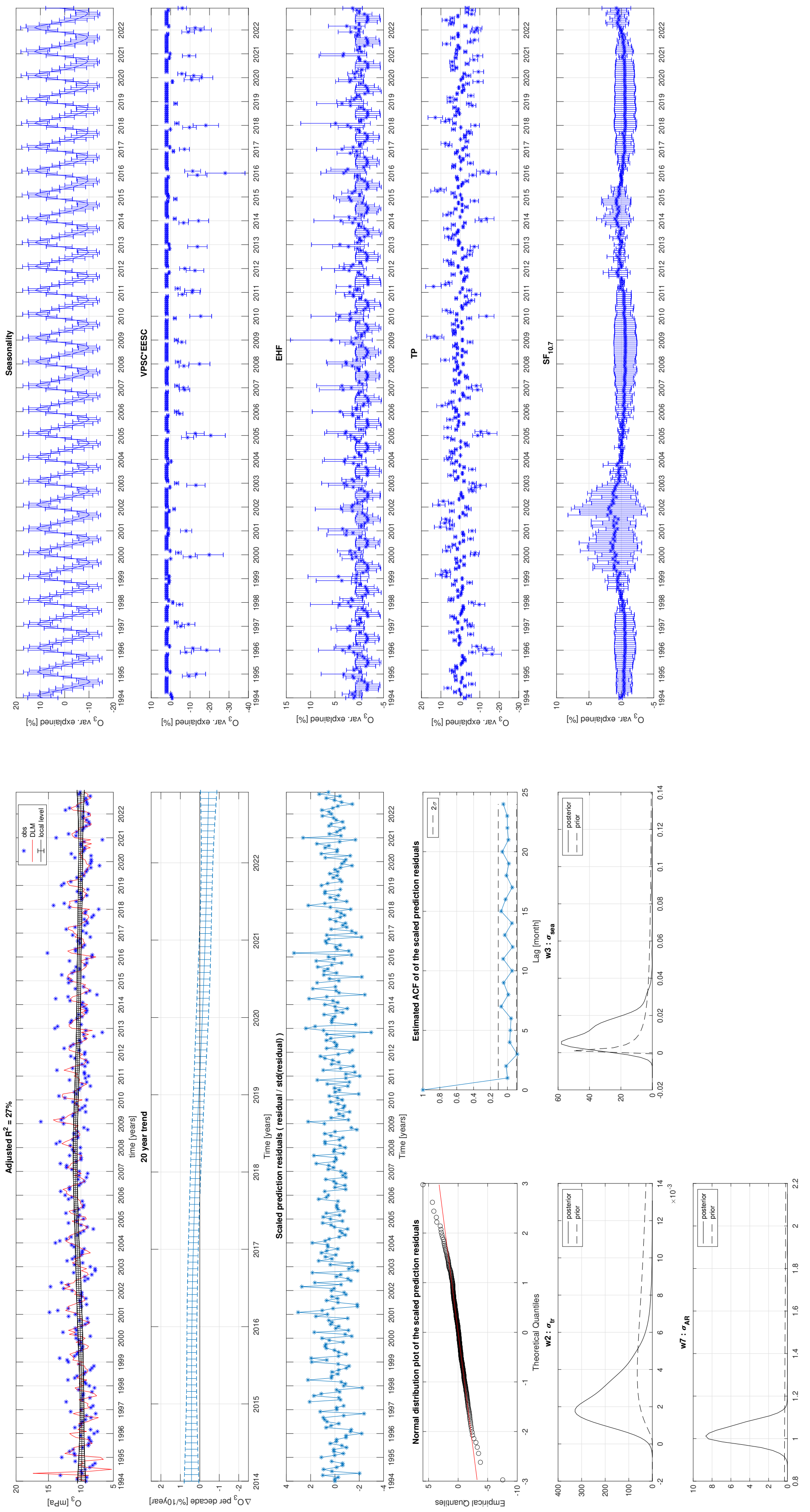

Figure S23. Ittoqqortoormiit (Scoresbysund) at L_4 altitude layer

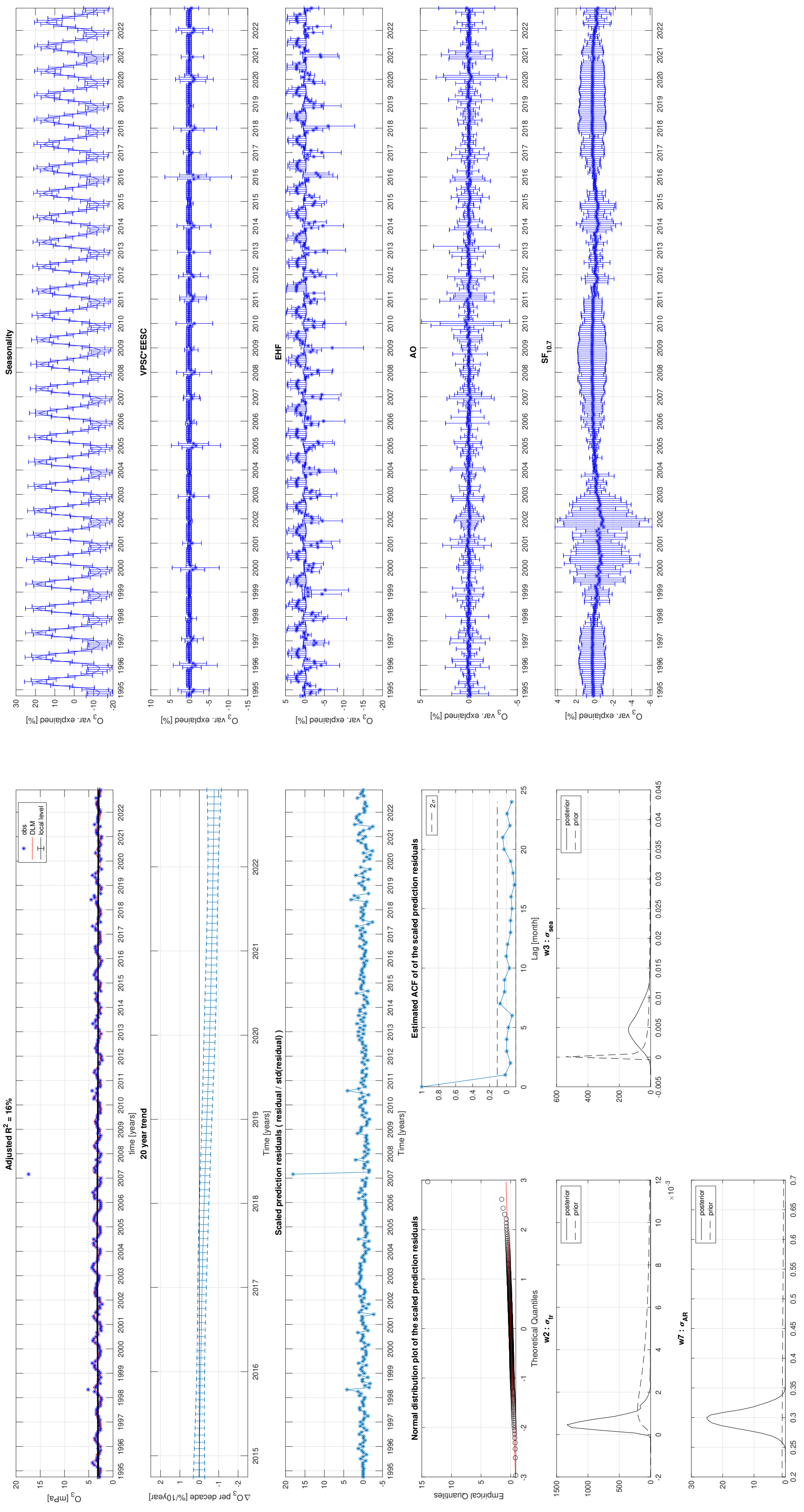

Figure S24. Sodankylä at L_1 altitude layer

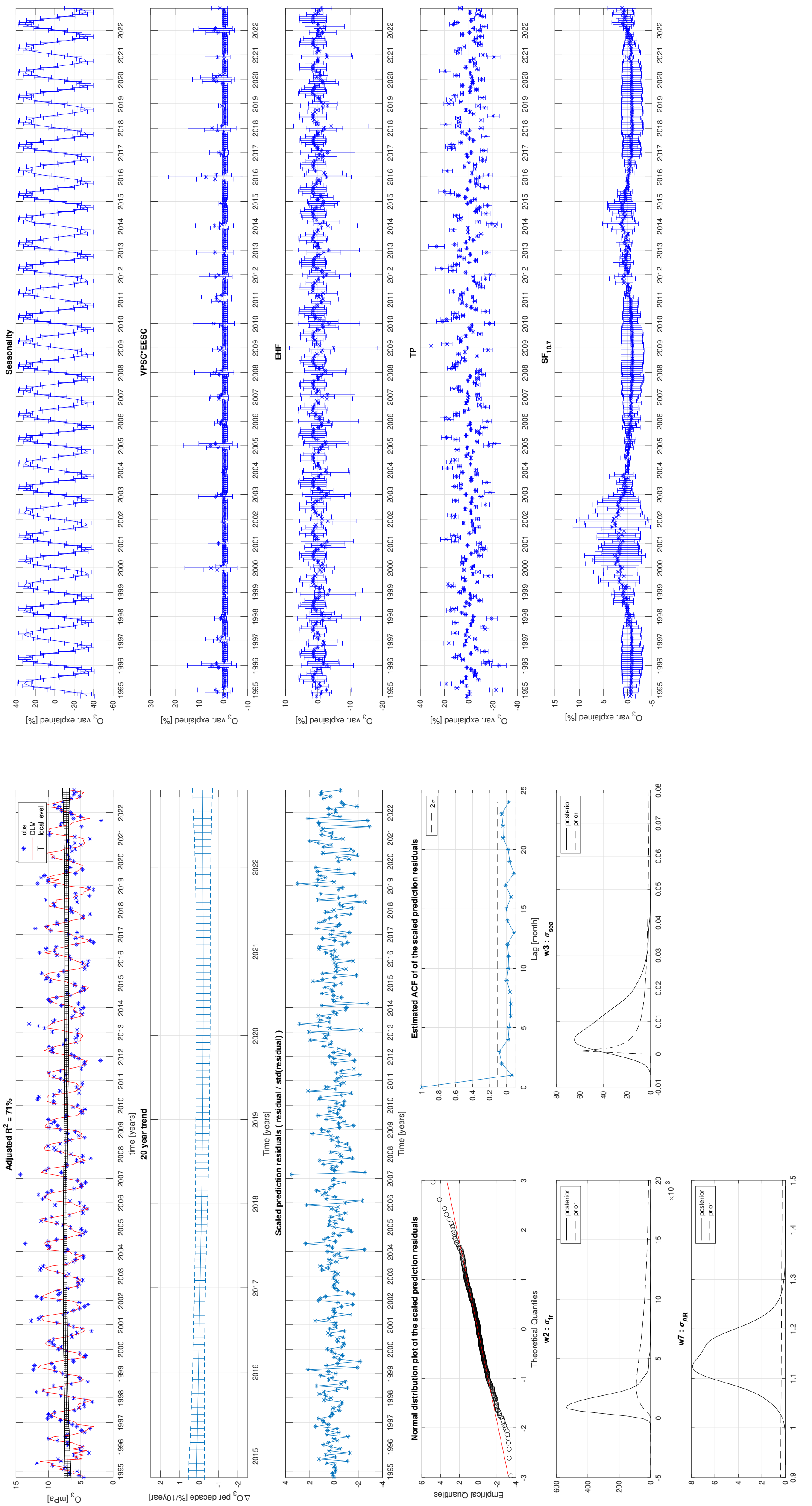

Figure S25. Sodankylä at L_2 altitude layer

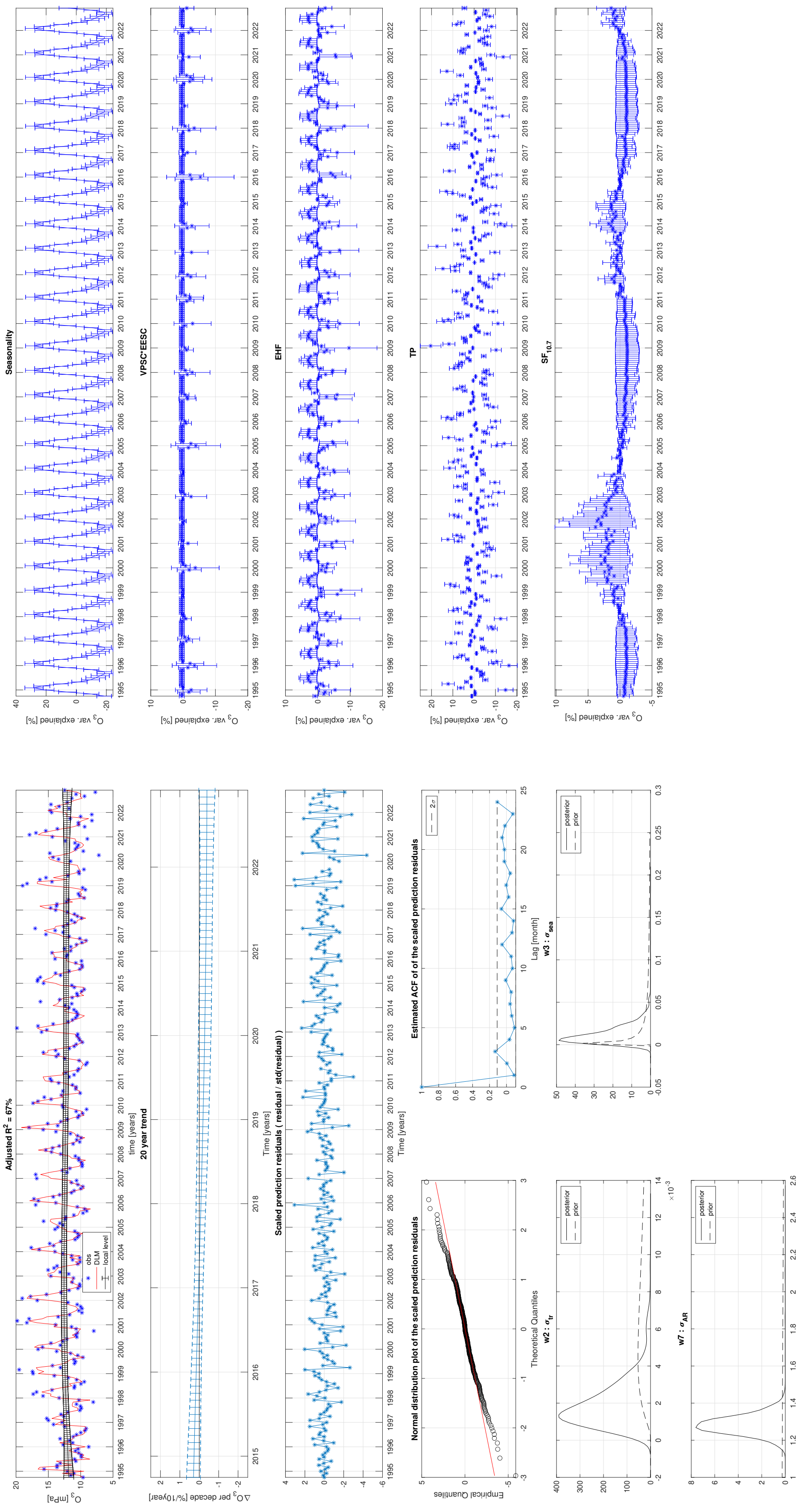

Figure S26. Sodankylä at L_3 altitude layer

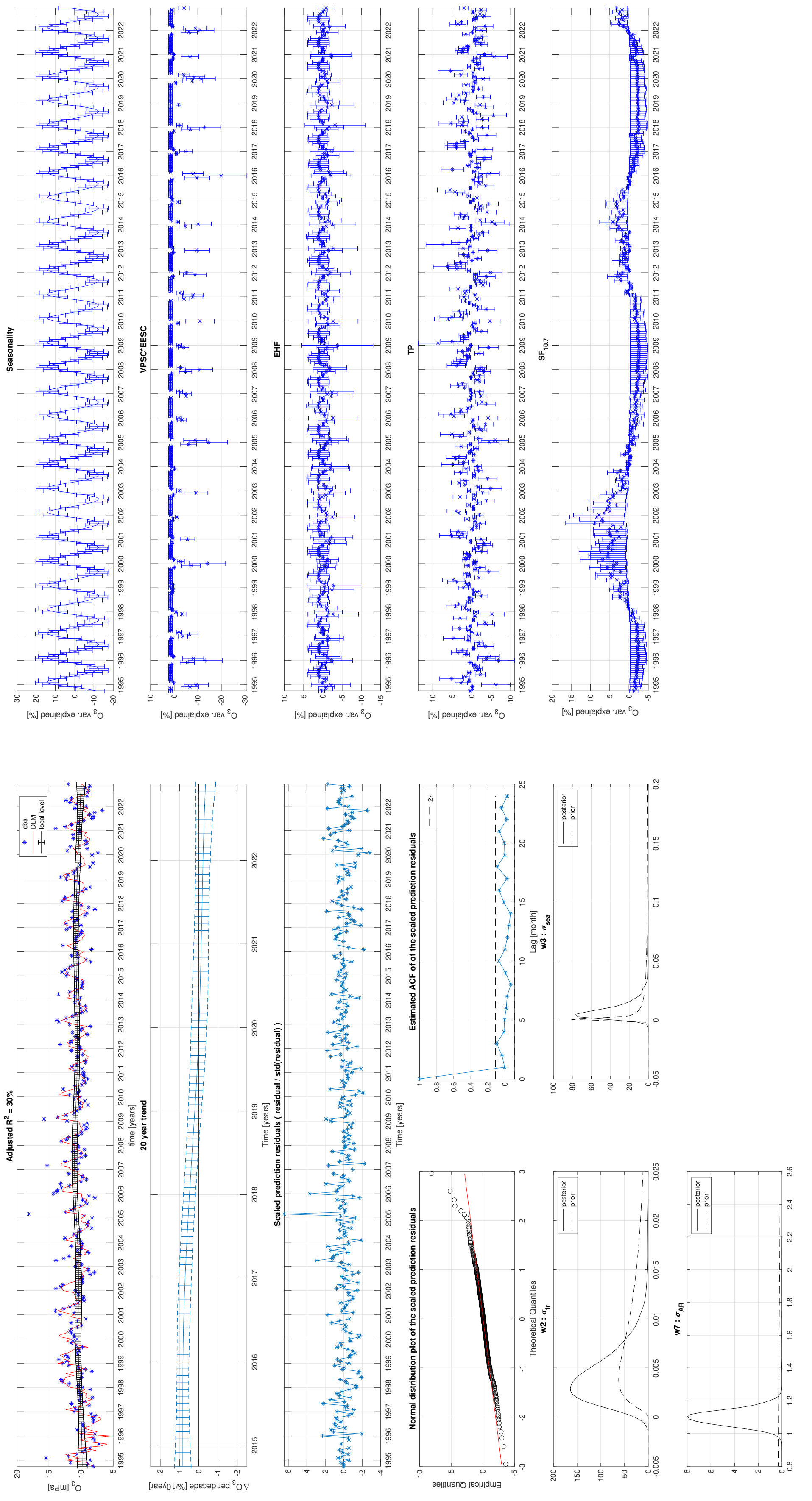

Figure S27. Sodankylä at L_4 altitude layer
